# International consensus validation of the POPI tool (Pediatrics: Omission of Prescriptions and Inappropriate prescriptions) to identify inappropriate prescribing in pediatrics

**DOI:** 10.1371/journal.pone.0240105

**Published:** 2020-10-05

**Authors:** Laily Sadozai, Shaya Sable, Enora Le Roux, Pierre Coste, Clémence Guillot, Priscilla Boizeau, Aurore Berthe-Aucejo, François Angoulvant, Mathie Lorrot, Olivier Bourdon, Sonia Prot-Labarthe

**Affiliations:** 1 Pharmacy Department, Robert-Debré Hospital, AP-HP, Paris, France; 2 Unité d’épidémiologie clinique, Hôpital Universitaire Robert Debré, AP-HP.Nord-Université de Paris, Inserm, CIC 1426, Paris, France; 3 ECEVE UMR 1123, Université de Paris, Inserm, Paris, France; 4 Necker Hospital, AP-HP, Paris, France; 5 Trousseau Hospital, AP-HP, Paris, France; 6 Clinical Pharmacy, Paris Descartes University, Paris, France; 7 Education and Health Practices, Paris XIII University, Bobigny, France; 8 Pediatric Group, Société Française de Pharmacie Clinique, Paris, France; University of South Australia, AUSTRALIA

## Abstract

**Introduction:**

While drug prescription should be based on established recommendations stemming from clinical trials but in pediatrics, many drugs are used without marketing authorization. Consequently recommendations are often based on clinical experience and the risk of inappropriate prescription (IP) is high. A tool for detecting IP in pediatrics—called POPI (Pediatrics: Omission of Prescriptions and Inappropriate prescriptions)—has been developed in France. However the relevance of its use at an international level is not known. Our aim has been to adapt POPI for a worldwide use.

**Material and method:**

A two-round Delphi online questionnaire was completed and validated by international experts to identify consensual items. They were asked to rate the validity of each items taking into account the recommendations and practices in their countries. Only propositions obtaining a median score in the upper tertile with an agreement of more than 75% of the panel—for the first round—and 85%—for the second round—were retained.

**Results:**

Our panel included 11 pharmacists (55%) and 9 physicians (45%). The panelists came from 12 different countries: England, Belgium, Brazil, Canada, China, Ivory Coast, Ireland, Malaysia, Portugal, Switzerland, Turkey and Vietnam. At the end of the first round, of the 105 items of the original POPI tool, 80 items were retained including 16 items reworded and 25 items were deleted. In the second round, 14 experts participated in the study. This final international POPI tool is composed of 73 IP and omissions of prescriptions in the fields of neuropsychiatry, dermatology, infectiology, pneumology, gastroenterology, pain and fever.

**Discussion and conclusion:**

This study highlights international consensus on prescription practice in pediatrics. The use of this tool in everyday practice could reduce the risk of inappropriate prescription. The impact of the diffusion of POPI tool will be assessed in a prospective multicentric study.

## Introduction

Drug prescription for newborns, infants, and children is often based on measures of drug safety and effectiveness assessed in adults [1]. Many drugs are used off-label or without marketing authorization (MA) [2]. The use of medication in pediatrics should be based on established recommendations from well-conducted clinical trials; however, in the absence of such trials, recommendations are often based on clinical experience. Misuse of medications can lead to adverse drug reaction (ADR) in pediatrics where the heterogeneous population undergoes important pharmacokinetics changes [3]. Incidence of ADR leading to hospital admission has been evaluated between 1.8% and 17.7% [4, 5]. The elderly population, frequently polymedicated is also at high risk of ADR. Many tools have been developed to detect inappropriate prescriptions (IP) in this population: the *Beers Criteria for Potentially Inappropriate Medication Use in Older Adults*, the STOPP/START (*Screening Tool of Older Person's Prescriptions* / *Screening Tool to Alert doctors to Right Treatment*) [6, 7]. This is largely due to the susceptibility of the elderly to become ill and it is also due to the prevalence of polypharmacy in this population. In a 2008 study, the use of the STOPP list identified IPs in 35% of a cohort of elderly patients; one third of these IPs were associated with an adverse drug event [8]. Another study involving randomized hospitalized patients showed that the occurrence of IP was 35% lower in patients who were prescribed drugs according to STOPP/START criteria than in patients for whom usual pharmaceutical criteria were used [9]. Pediatric prescription is also in need of additional securing; for this reason, a first IP tool in pediatrics called POPI (Pediatrics: Omission of Prescriptions and Inappropriate prescriptions) has been developed in France. POPI was composed of a list of inappropriate prescriptions and omissions linked to pediatric health problems, identified in 2010 according to the frequency of hospitalization related to them and their prevalence according to pediatric data extracted from the French National Health Insurance Fund for Employees. For each of the chosen pediatric health problems, recommendations that were both backed up by evidence and published after 2000 were identified. Based on these recommendations, a consensual survey allowed us to develop the POPI tool [10].

However, POPI was constructed to satisfy French standards, established by national experts, with a strong reliance on French databases, drug availability and unique problematics. Since the need for such a tool has been identified internationally, our aim is to adapt POPI at the international level, using health professionals from different countries to identify which items could be of worldwide use.

## Material and methods

### Study design

An online consensus survey has been completed using the Delphi method. It consists of two or more questionnaire rounds that are completed to achieve a consensus among a panel of experts. It implies a feedback process in which group responses obtained during one round, are returned to the participants during the next round in the form of statistical summaries [11]. A two round Delphi was executed to identify the items of the original POPI applicable internationally.

The original POPI contained 104 items. We then removed three propositions because new recommendations were published. At this stage, the tool contained 101 propositions. Some of the 101 items of the initial French POPI tool were split into two sentences to augment clarity. The tool, now composed of 105 items and was used as a starting point for the international version [10] (Table A in S1 Table). Ethical approval for this study was obtained from the local ethic committee of the Robert Debré hospital, Paris, France (number 2016/343).

### Selection and recruitment of the Delphi panel

Several methods of recruitment were used to select our panel. Our experts include hospital pharmacists and pediatrician who read the previous POPI publication [10] and showed interest in the tool, members of the European Society of Clinical Pharmacy as well as researchers working on similar topics. The latter were approached after we selected publications on PubMed using « pediatric medication safety » mesh terms from February 2019 and over the past 5 years, then inviting the publications’ authors to participate in our study. Experts were pediatricians and pharmacists with a median of 20 years of experience (from 5 to 20 years).

### Data collection and criteria of analysis

To be recruited, participants were asked by e-mail to return a declaration of participation and absence of conflict of interest then a e-link was sent to them in order to connect to the secure survey site Experts were asked to rate the validity and feasibility of items concerning prescriptions on a 9-point Likert scale, with 1 being « definitely not valid or not feasible [in my country] » and 9 « definitely valid and feasible [in my country] ». They were encouraged to make suggestions about dosage, frequency, and duration of treatment, and to provide appropriate references. Justifications on the non-validity or non-feasibility of items were required for a score under 7/9. Indeed, experts had to choose between three different statements: (1) “The proposition is accepted as it is, and does not need modification”, (2) “The proposition is accepted but needs modification (specify below)” or (3) “The proposition is not accepted (justify below)”. The definition of the agreement was the number of criteria (1) and (2) above the total number of responses for each item.

For the first round, we discarded items according to the following cutoffs: median score less than 7/9 or an agreement of less than 75% among the entire panel of experts, or a rejection by 25% of participating countries for national drug unavailability/national recommendations contraindications. Agreement corresponded to the proportion of scores over 7/9. For each question, individual answers of the first round and the median score of the entire panel were mentioned. For the second round, the health care professionals had to confirm or refute their results knowing the panel’s global answer. Selection criteria was different from the first round with a restricting agreement of 85% in addition to a median score superior to 7/9.

To study the impact of missing data on the results of the second round a sensitivity analysis was made. It compared the final results if the participation rate was the same between the first and the second round, under the assumption that the participants answers did not change between the two rounds. To do that, we replaced the missing answers of the expert in the second with their answer of the first round.

## Results

In the first round of the Delphi process, 20 experts representing 12 different countries took part to the POPI worldwide project. Eleven (55%) of them were pharmacists and 9 (45%) of them were pediatricians. The participants formed a panel with 5 to 40 years of experience (Table 1).

**Table 1:** Demographic data about the 20 experts.

Variables	Median (min—max) or N (%)
**Age (year)**	45 (28–64)
**Years of experience**	20 (5–40)
**Pharmacists**	11 (55%)
**Doctors**	9 (45%)
**Number of experts per country–N (%)**	
**Belgium**	1 (5%)
**Brazil**	2 (10%)
**Canada**	2 (10%)
**China**	1 (5%)
**England**	2 (10%)
**Ireland**	1 (5%)
**Ivory Coast**	1 (5%)
**Malaysia**	2 (10%)
**Portugal**	2 (10%)
**Switzerland**	2 (10%)
**Turkey**	2 (10%)
**Vietnam**	2 (10%)

**Fig 1:**
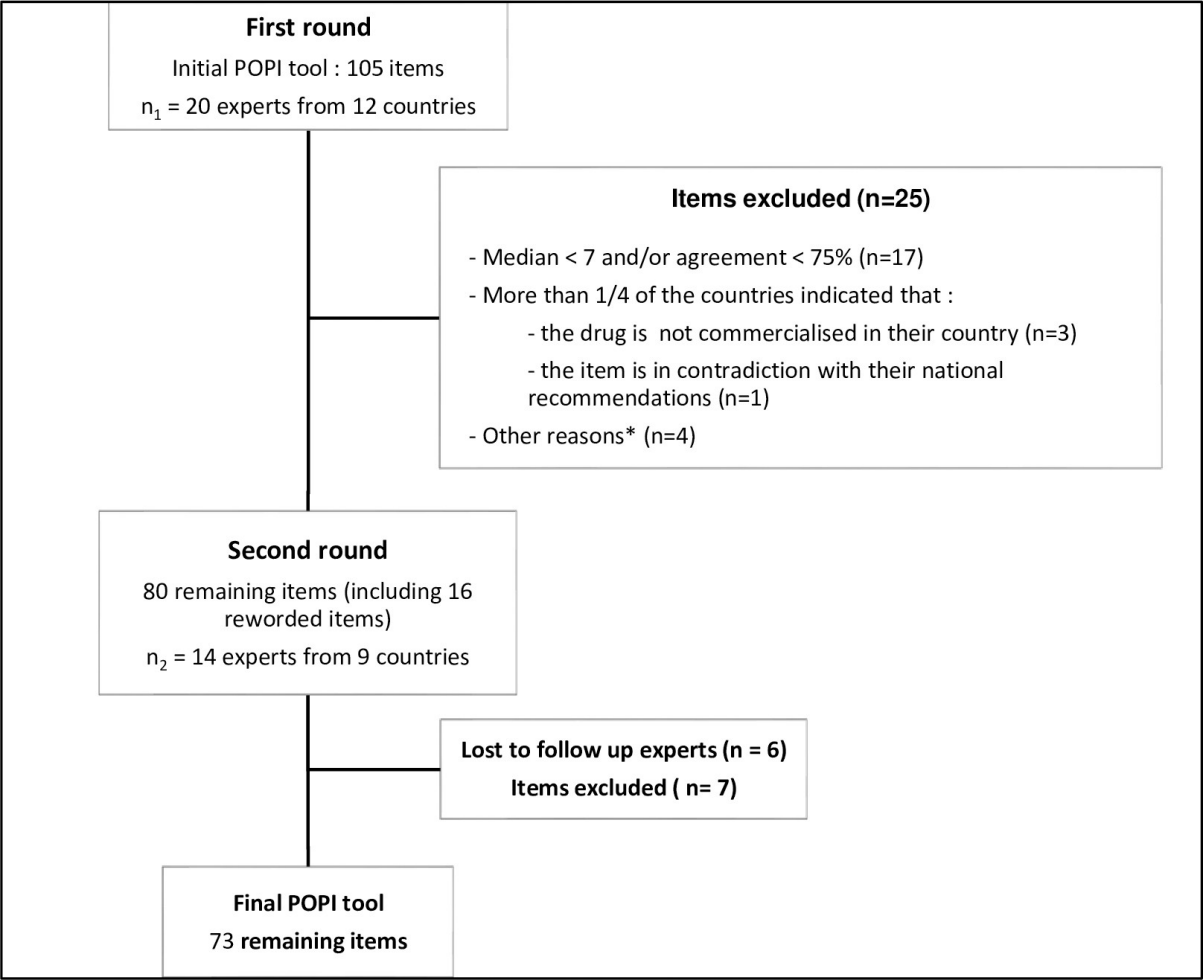
Two rounds Delphi process flowchart. *The four other reasons for removal refers to: (1) The item about ibuprofen in the category “pain and fever” was specific to the French tool as it was referring to the different french commercial forms of the drug; (2) For the item “IP of alimemazine, oxomemazine and promethazine”in the category “Ent–pulmonary cough problems” several experts agreed with the item but the drugs were not available in many countries; (3) The item about palivizumab was removed because the doses and the age of prescription were different from one country to another even though the majority of the experts agreed with the palivizumab indication of prescription; (4) Regarding the item”application of topical antibiotic”, we proved that some topical antibiotics could be applied less than twice a day, such as fusidic acid cream.

The first round started in May 2018. Eighty items were rated in the upper tertile by more than 75% of the panel. In other words, 76% of the items were maintained and among them, 16 were reworded after the panel’s suggestions (Fig 1). This first round allowed us to gather 150 comments about practices in each country. Among all the items, 25 were excluded from the tool as they couldn’t be applied by all the countries. Indeed, drugs in question were either not commercialized in their country or the proposition was deemed in contradiction with their local recommendations. For the second round, 14 experts from 9 countries (70%) participated again, while six experts from Brazil, Canada, China, England and Ireland desisted. The second round started in March 2019. By reducing the agreement to 85%, we finally obtained 73 items (Table 2), with one item reworded. Thirty-three items (45% of the final tool) obtained 100% agreement among the entire panel. According to the sensitivity analysis, if the participation rate at the second round was similar to the first one, the final POPI tool would be composed of 66 items– 7 supplementary items wouldn’t reach the 85% agreement (Table B in S1 Table).

**Table 2:** Description of the final POPI tool (n = 73 items).

Item	1^st^ round Median (min–max)	1^st^ round Agreement (%)	2^nd^ round Median (min–max)	2^nd^ round Agreement (%)
**Diverse pain and fever illnesses**				
**Inappropriate prescription**				
Prescription of two alternating antipyretics as a first-line treatment	9 (1 – 9)	89.5	9 (7–9)	100
Prescription of a medication other than acetaminophen/paracetamol as a first line treatment (except in the case of migraine)	9 (2 – 9)	94.4	9 (3–9)	92.9
Rectal administration of paracetamol as a first-line treatment	8 (1 – 9)	78.9	8.5 (1–9)	85.7
The combined use of two NSAIDs	9 (1 – 9)	94.4	9 (7–9)	100
Opiates to treat migraine attacks	9 (3 – 9)	89.5	9 (8–9)	100
**Omission of prescription**				
Failure to give sugar solution to new-born babies and infants under four months old two minutes prior to venipuncture	9 (2 – 9)	88.9	9 (8–9)	100
Failure to give an osmotic laxative to patients being treated with morphine for a period of more than 48 hours	8 (2 – 9)	82.4	9 (8–9)	100
**Urinary infections**				
**Inappropriate prescription**				
Antibiotic prophylaxis following an initial infection without complications (except in the case of uropathy)	9 (1 – 9)	94.7	9 (9–9)	100
Antibiotic prophylaxis in the case of asymptomatic bacterial infection (except in the case of uropathy)	9 (1–9)	94.7	9 (8–9)	100
**Vitamin supplements and antibiotic prophylaxis**				
**Inappropriate prescription**				
Fluoride supplements prior to six months of age	9 (1–9)	94.7	9 (9–9)	92.9
**Mosquitos**				
**Inappropriate prescription**				
Citronella (lemon grass) essential oil	9 (1–9)	82.4	8.5 (1–9)	85.7
Anti-insect bracelets to protect against mosquitos and ticks	9 (1–9)	83.3	9 (1–9)	85.7
Ultrasonic pest control devices, vitamin B1, homeopathy, electric bug zappers, sticky tapes without insecticide	9 (1–9)	88.2	9 (1–9)	85.7
**Omission of prescription**				
Mosquito nets and clothes treated with pyrethroids	9 (1–9)	83.3	9 (1–9)	85.7
**Digestive nausea, vomiting or gastroesophageal reflux problems**				
**Inappropriate prescription**				
Metoclopramide	9 (3–9)	94.4	9 (3–9)	92.9
Domperidone	8 (2–9)	77.8	8.5 (3–9)	85.7
Oral administration of an intravenous proton pump inhibitor (notably by nasogastric tube)	9 (1–9)	94.7	9 (8–9)	100
Gastric antisecretory drugs to treat gastroesophageal reflux, dyspepsia, the crying of new-born babies (in the absence of any other signs or symptoms), as well as faintness in infants	9 (1–9)	94.7	9 (6–9)	92.9
The combined use of proton pump inhibitors and NSAIDs, for a short period of time, in patients without risk factors	9 (3–9)	94.7	9 (8–9)	100
The use of type H2 antihistamines for long periods of treatment	9 (4–9)	100	9 (8–9)	100
Oral rehydration solution	9 (7–9)	100	9 (5–9)	85.7
**Diarrhea**				
**Inappropriate prescription**				
Loperamide before 3 years of age	9 (1–9)	84.2	9 (6–9)	85.7
Loperamide in the case of invasive diarrhea	9 (1–9)	94.7	9 (9–9)	100
The use of Saccharomyces boulardii in powder form, or in a capsule that has to be opened prior to ingestion, to treat patients with a central venous catheter or an immunodeficiency	9 (1–9)	89.5	9 (8–9)	100
Intestinal antiseptics	9 (1–9)	94.4	9 (9–9)	100
**Omission of prescription**				
Oral rehydration solution	9 (1–9)	100	9 (6–9)	85.7
**ENT-Pulmonary cough problems**				
**Inappropriate prescription**				
Mucolytic drugs, mucokinetic drugs, or helicidine before two years of age	9 (1–9)	78.9	9 (1–9)	92.9
**Bronchiolitis in infants**				
**Inappropriate prescription**				
Beta2 agonists, corticosteroids to treat an infant’s first case of bronchiolitis	9 (1–9)	94.7	9 (6–9)	92.9
H1-antagonists, cough suppressants, mucolytic drugs, or ribavirin to treat bronchiolitis	9 (1–9)	94.7	9 (9–9)	100
Antibiotics in the absence of signs indicating a bacterial infection (acute otitis media, fever, etc.)	9 (1–9)	94.7	9 (9–9)	100
**Omission of prescription**				
0.9% NaCl to relieve nasal congestion (not applicable if nasal congestion is already being treated with 3% NaCl delivered by a nebulizer)	9 (1–9)	89.5	9 (8–9)	100
**ENT infections**				
**Inappropriate prescription**				
An antibiotic other than amoxicillin as a first-line treatment for acute otitis media, strep throat, or sinusitis (provided that the patient is not allergic to amoxicillin). An effective dose of amoxicillin for an pneumoncoccal infection is 80–90 mg/kg/day and an effective dose for a streptococcal infection is 50 mg/kg/day	9 (5–9)	94.4	9 (7–9)	100
Antibiotic treatment for a sore throat, without a positive rapid diagnostic test result, in children less than three years old	9 (1–9)	88.9	9 (5–9)	92.9
Antibiotics for–nasopharyngitis-congestive otitis-sore throat before three years of age-laryngitis-as a first-line treatment for acute otitis media showing few symptoms, before two years of age	9 (3–9)	82.4	9 (5–9)	92.9
Antibiotics to treat otitis media with effusion (OME), except in the case of hearing loss or if OME lasts for more than three months	9 (1–9)	84.2	9 (7–9)	100
Corticosteroids to treat acute suppurative otitis media, nasopharyngitis, or strep throat	9 (1–9)	94.7	9 (8–9)	92.9
Nasal or oral decongestant (oxymetazoline, pseudoephedrine, naphazoline, ephedrine, tuaminoheptane, phenylephrine)	9 (4–9)	82.4	9 (5–9)	92.9
H1-antagonists with sedative or atropine-like effects (pheniramine, chlorpheniramine), or camphor; inhalers, nasal sprays, or suppositories containing menthol (or any terpene derivatives) before 30 months of age	9 (1–9)	84.2	9 (8–9)	100
Ear drops in the case of acute otitis media	9 (5–9)	100	9 (7–9)	100
**Omission of prescription**				
Acetaminophen/paracetamol combined with antibiotic treatment for ear infections to relieve pain	9 (1–9)	89.5	9 (9–9)	100
**Asthma**				
**Inappropriate prescription**				
Ketotifen and other H1-antagonists, sodium cromoglycate	9 (1–9)	78.9	9 (5–9)	92.9
Cough suppressants	9 (1–9)	94.4	9 (9–9)	100
**Omission of prescription**				
Asthma inhaler appropriate for the child’s age	9 (8–9)	100	9 (9–9)	92.9
Preventative treatment (inhaled corticosteroids) in the case of persistent asthma	9 (7–9)	94.7	9(9–9)	92.9
**Dermatological acne vulgaris problems**				
**Inappropriate prescription**				
Isotretinoin in combination with a member of the tetracycline family of antibiotics	9 (5–9)	94.1	9 (7–9)	92.9
The combined use of an oral and a local antibiotic	9 (5–9)	100	9 (8–9)	92.9
Oral or local antibiotics as a monotherapy (not in combination with another drug)	9 (1–9)	83.3	9 (8–9)	92.9
**Scabies**				
**Omission of prescription**				
A second dose of ivermectin two weeks after the first	9 (1–9)	89.5	9 (1–9)	85.7
Decontamination of household linen and clothes and treatment for other family members	9 (8–9)	94.7	9 (8–9)	100
**Ringworm**				
**Omission of prescription**				
Topical treatment combined with an orally-administered treatment	9 (3–9)	88.9	9 (3–9)	85.7
**Impetigo**				
**Inappropriate prescription**				
The combination of locally applied and orally administered antibiotic	9 (6–9)	94.7	9 (8–9)	92.9
**Herpes simplex**				
**Inappropriate prescription**				
Topical agents containing corticosteroids	9 (1–9)	94.7	9 (9–9)	92.9
**Omission of prescription**				
Acetaminophen/paracetamol during an outbreak of herpes	9 (5–9)	94.7	9 (5–9)	85.7
Orally administered acyclovir to treat primary herpetic gingivostomatitis	9 (1–9)	84.2	9 (8–9)	92.9
**Atopic eczema**				
**Inappropriate prescription**				
A strong dermocorticoid (clobetasol propionate 0.05% Dermoval, betamethasone dipropionate Diprosone) applied to the face, the armpits or groin, and the backside of babies or young children.	9 (4–9)	100	9 (9–9)	100
More than one application per day of a dermocorticoid, except in cases of severe lichenification	9 (1–9)	77.8	9 (2–9)	92.9
Local or systemic antihistamine during the treatment of outbreaks	8 (1–9)	83.3	9 (6–9)	92.9
Topically applied 0.03% tacrolimus before two years of age	9 (4–9)	94.4	9 (7–9)	100
Topically applied 0.1% tacrolimus before 16 years of age	9 (1–9)	84.2	9 (5–9)	92.9
Oral corticosteroids to treat outbreaks	8 (4–9)	83.3	9 (5–9)	92.9
**Neuropsychiatric epilepsy disorders**				
**Inapproproate prescription**				
Carbamazepine, gabapentin, oxcarbazepine, phenytoin, pregabalin, tiagabine, or vigabatrin in the case of myoclonic epilepsy	9 (1–9)	94.7	9 (9–9)	92.9
Carbamazepine, gabapentin, oxcarbazepine, phenytoin, pregabaline, tiagabine, or vigabatrin in the case of epilepsy with absence seizures (especially for childhood absence epilepsy or juvenile absence epilepsy)	9 (2–9)	89.5	9 (9–9)	92.9
**Depression**				
**Inappropriate prescription**				
Tricyclic antidepressants to treat depression	9 (2–9)	89.5	9 (7–9)	92.9
**Nocturnal enuresis**				
**Inappropriate prescription**				
Desmopressin administered by a nasal spray.	8 (1–9)	83.3	9 (1–9)	92.9
Desmopressin in the case of daytime symptoms	9 (1–9)	94.7	9 (8–9)	100
An anticholinergic agent used as a monotherapy in the absence of daytime symptoms	9 (1–9)	94.7	9 (7–9)	100
Tricyclic agents in combination with anticholinergic agents	9 (1–9)	89.5	9 (6–9)	92.9
Tricyclic agents as a first-line treatment	9 (1–9)	94.7	9 (8–9)	100
**Anorexia**				
**Inappropriate prescription**				
Cyproheptadine, clonidine	9 (2–9)	88.9	9 (5–9)	85.7
**Attention deficit disorder with or without hyperactivity**				
**Inappropriate prescription**				
Pharmacological treatment before age six (before school), except in severe cases	9 (2–9)	94.7	9 (8–9)	100
Antipsychotic drugs to treat attention deficit disorder without hyperactivity	9 (1–9)	89.5	9 (8–9)	100
Slow release methylphenidate as two doses per day, rather than only one dose	9 (1–9)	94.7	9 (9–9)	100
**Omission of prescription**				
Recording a growth chart (height and weight) if the patient is taking methylphenidate	9 (1–9)	89.5	9 (9–9)	100

## Discussion

From the original French version of POPI, 32 items were removed. It is a first step in adopting this tool of inappropriate prescribing and omission of prescription in pediatrics for international use.

This method of tool adaptation has already been carried out in the field of inappropriate specifications: the STOPP and START criteria were initially validated through a Delphi process in Ireland and United Kingdom. An evaluation of reliability between multiple physicians across six European centers was needed to generalize the STOPP and START criteria [12]. In our case, inter-rater reliability has been evaluated by eleven clinicians with no previous experience of the tool [13]. Our methodology to spread the use of the POPI tool was a Delphi process including physicians and pharmacists into our panel of experts.

The most common reason for the dismissal of an item was a contradiction with national recommendations. One of the examples was the use of nitrofurantoin. Indeed, antibiotic guidelines differed from one country to another due to the local bacterial resistance and antibiotic availability. French product information indicated that nitrofurantoin is contraindicated for children under 6 years old whether used as a prophylactic or curative treatment [14], because of its hepatic and pulmonary side effects, but also because of the pharmaceutical forms available in France. However, this item was discarded from the POPI tool as 8 countries (66%) used nitrofurantoin as a prophylactic or curative antibiotic in pediatric urinary tract infections [15].

The second most common reason was drug unavailability. When drugs were unavailable, the idea of rewording was discussed and eventually dismissed as the item’s worldwide use was deemed limited. This was the case for example for diosmectite unavailable in 6 of the 12 surveyed countries (50%) according to the panel of experts (Belgium, Brazil, England, Switzerland, Turkey and Canada). Recently, the French health agency (National Agency for the Safety of Medicines and Health Products—ANSM) published a warning concerning diosmectite use for diarrhea in pediatrics. Indeed, diosmectite is composed of clay and is now contraindicated for children under 2 years old in France, due to the presence of a small amount of lead particles which are more likely to pass into the bloodstream of pediatric patients than that of adults.

Neurological and cardiovascular side effects of antiemetic drugs must also be considered. Indeed, the French drug agency (ANSM) published a warning in 2014 about domperidone [16]. This warning highlighted the risk of potential high cardiovascular side effects that can occur in adult and pediatric patients using domperidone [17, 18]. Because of these potentially serious side effects, domperidone pediatric prescriptions were no longer reimbursed by the French health care system after 2017. Following this Delphi process, we found that several experts from different countries suggested that domperidone is still used in pediatric patients, especially for gastroesophagial reflux or for short time administration. Countries such as Belgium, England, Ireland, Malaysia, Switzerland and Vietnam still use domperidone. Nevertheless, the final score of the Delphi process allowed us to maintain this item in the POPI tool.

Another antiemetic drug was the subject of a controversy. We suggested that metoclopramide prescription was inappropriate in children. Metoclopramide is a dopamine-2 antagonist known for its neurological and cardiovascular side effects such as dyskinesia disorders [19–22]. In France, metoclopramide is contraindicated in patient under 18 year old, and has been since 2012 [23]. This contraindication may lead to an increase of domperidone prescription and consumption, but as we mentioned earlier, domperidone is also an inappropriate prescription according to the POPI tool.

Another common disagreement was in criteria of inclusion for drug treatment. Our item regarding the minimum vitamin D intake, needed in children for bone growth, was met with a strong disapproval. The main point of contention was dosage and patient inclusion: while some countries recommend a standardized dose, regardless of patient’s diets or prematurity, others ponder these factors and adjust vitamin intake accordingly. Others, mostly very sunny countries, don’t recommend any supplementation [24].

These disparate criteria of inclusion have impact on another one of our items: whooping cough booster injections. France recommends it for young adults, arriving at common child-bearing years, regardless of sex [25]. Some countries recommend these shots to women or pregnant women to protect the future neonate, while some have no national guidelines regarding adult DTPca shots. On this topic, our experts conceded their national guidelines were based on economic reasoning rather than scientific consensuses.

There is a strong demand for increased pediatric medication safety [26–28]. POPI, validated bu 20 experts–both pharmacists and physicians recruited across the world, it the first international tool created to satisfy this demand, by the detection of pediatric inappropriate prescription and omission of prescription. The other strength of this study is that nearly half of the final POPI tool reached a complete consensus among the panel of experts. This is strong evidence of harmonization of the item and a proof that the tool can be applied in all the participating countries. It is also the reason why two rounds of the Delphi process were enough to reach a consensus.

Nevertheless, we could have benefited from a larger group of panelists. We originally intended to recruit a duo of pharmacist/physician in each country: we discarded the idea for its lack of feasibility. The length of the survey as well as the personal implication needed could have discouraged some of the experts contacted. In addition, some of the world’s largest players in health care are missing, notably the USA, however this can be made up for by the diversity of our participants.

Regarding the expert’s selection, the only criteria to be accepted into the study was their motivation to participate. Few information was known about their skills or competences apart their year of experience. We cannot impose a minimal year of experience as it goes against the Delphi method. The Delphi process tends to draw a consensus between an heterogenous and large panel of experts. The majority of the disagreement answers were justified with national recommendations, publications or comments that were carefully analyzed.

There were also too few experts per country to determine whether there is anything resembling to a "within country" consensus. It was only an international consensus among countries that are quite heterogeneous in their prevalence of diseases and access to resources. We could compare north versus south countries responses however this kind of comparison would raise differences instead of consensus.

It is sometimes hard to grade both validity and feasibility of each item in each country has it is two different things. For example, the experts can totally agree with an item but it sometimes cannot be applicable in their country because of several reasons. It was primordial to analyze both feasibility and validity to have a complete interpretation but it can also be a methodologic limitation of our study.

Other pediatric tools and indicators are also emerging such as the Potentially Inappropriate Prescribing indicator in children (PIPC) [29]. It consists of twelve indicators concerning primary care settings in Ireland and United Kingdom. England also adapted the original POPI tool for their practices [30]. The methodology was different, as they didn’t refer to a panel of experts: one researcher compared the items to national guidelines. After that, 80 items were remained. It is interesting to see that there is a similarity between the items removed in this project and in POPI international.

There is a lack of established recommendations in pediatrics and a high risk of inappropriate prescription. For this reason, a prospective multicentric study evaluating the impact of applying POPI to several countries should be implemented. This approach in other countries will allow us to see whether the tool works as well as it did in France and to identify potential issues in implementation in health systems other than the French one.

## Conclusion

The harmonization by 20 international experts of the POPI tool allowed us to confirm the validity of the first inappropriate prescription and omission of prescription tool in pediatrics. Indeed, spreading POPI’s use would be an opportunity to help pediatricians and pharmacists to reduce potentially dangerous inappropriate prescriptions in pediatrics. The next step would be a prospective multicentric study in order to promote pediatric medication safety into many countries.

## Supporting information

S1 Table (DOCX)
