# Supplementary material for: International consensus validation of the POPI tool (Pediatrics: Omission of Prescriptions and Inappropriate prescriptions) to identify inappropriate prescribing in pediatrics
Source: PLoS One. 2020 Oct 5;15(10):e0240105. doi: 10.1371/journal.pone.0240105 (PMC7535059; doi:10.1371/journal.pone.0240105)
Supplement: S1 Table — (DOCX) [file pone.0240105.s001.docx]

**Table A: Initial POPI tool (n=105 items)**

| POPI’s items | Decision |
| --- | --- |
| DIVERSE PAIN AND FEVER ILLNESSES |  |
| IP: Prescription of two alternating antipyretics as a first-line treatment | **Maintained** |
| IP: Prescription of a medication other than acetaminophen/paracetamol as a first line treatment (except in the case of migraine) | **Maintained** |
| IP: Rectal administration of paracetamol as a first-line treatment | **Reworded** |
| IP: The combined use of two NSAIDs | **Maintained** |
| IP: Oral solutions of ibuprofen administered in more than three doses per day using a graduated pipette of 10mg/kg (other than Advil®) | **Refused** |
| IP: Opiates to treat migraine attacks | **Maintained** |
| OP: Failure to give sugar solution to new-born babies and infants under four months old two minutes prior to venipuncture | **Reworded** |
| OP: Failure to give an osmotic laxative to patients being treated with morphine for a period of more than 48 hours | **Reworded** |
| URINARY INFECTIONS |  |
| IP: Nitrofurantoin used as a prophylactic | **Refused** |
| IP: Nitrofurantoin used as a curative agent in children under six years of age, or indeed any other antibiotic if avoidable | **Refused** |
| IP: Antibiotic prophylaxis following an initial infection without complications (except in the case of uropathy) | **Reworded** |
| IP: Antibiotic prophylaxis in the case of asymptomatic bacterial infection (except in the case of uropathy) | **Maintained** |
| VITAMIN SUPPLEMENTS AND ANTIBIOTIC PROPHYLAXIS |  |
| IP: Fluoride supplements prior to six months of age | **Maintained** |
| OP: Insufficient intake of vitamin D. Minimum vitamin D intake: - Breastfed baby = 1 000 to 1 200 IU/day- Infant < 18 months of age (milk enriched in vitamin D) = 600 to 800 IU/day- Child aged between 18 months and five years, and adolescents aged between 10 and 18 years: two quarterly loading doses of 80 000 to 100 000 IU/day in winter (adolescents can take this dose in one go) | **Refused** |
| OP: Antibiotic prophylaxis with phenoxymethylpenicillin (Oracilline) starting from two months of age and lasting until five years of age for children with sickle-cell anemia: 100 000 IU/kg/day (in two doses) for children weighing 10kg or less and 50 000 IU/kg/day for children weighing over 10kg (also in two doses) | **Refused** |
| MOSQUITOS |  |
| IP: The use of skin repellents in infants less than six months old and picardin in children less than 24 months old | **Refused** |
| IP: Citronella (lemon grass) essential oil | **Maintained** |
| IP:  Anti-insect bracelets to protect against mosquitos and ticks | **Maintained** |
| IP:  Ultrasonic pest control devices, vitamin B1, homeopathy, electric bug zappers, sticky tapes without insecticide | **Maintained** |
| OP: Skin repellent- DEET concentrations- "30%" (max) before 12 years of age- "50%" (max) after 12 years of age | **Refused** |
| OP: Skin repellent- IR3535 concentrations- "20%" (max) before 24 months- "35%" (max) after 24 months | **Refused** |
| OP: Mosquito nets and clothes treated with pyrethroids | **Maintained** |
| DIGESTIVE NAUSEA, VOMITING OR GASTROESOPHAGEAL REFLUX PROBLEMS |  |
| IP: Metoclopramide | **Maintained** |
| IP: Domperidone | **Maintained** |
| IP: Oral administration of an intravenous proton pump inhibitor (notably by nasogastric tube) | **Maintained** |
| IP: Gastric antisecretory drugs to treat gastroesophageal reflux, dyspepsia, the crying of new-born babies (in the absence of any other signs or symptoms), as well as faintness in infants | **Reworded** |
| IP: The combined use of proton pump inhibitors and NSAIDs, for a short period of time, in patients without risk factors | **Maintained** |
| IP: The use of type H2 antihistamines for long periods of treatment | **Maintained** |
| IP: Erythromycin as a prokinetic agent | **Refused** |
| IP: The use of setrons (5-HT3 antagonists) for chemotherapy-associated nausea and vomiting | **Refused** |
| OP: Oral rehydration solution | **Maintained** |
| DIARRHEA |  |
| IP: Loperamide before 3 years of age | **Reworded** |
| IP: Loperamide in the case of invasive diarrhea | **Reworded** |
| IP: The use of Diosmectite in combination with another medication | **Refused** |
| IP: The use of Saccharomyces boulardii in powder form, or in a capsule that has to be opened prior to ingestion, to treat patients with a central venous catheter or an immunodeficiency | **Maintained** |
| IP: Intestinal antiseptics | **Maintained** |
| OP: Oral rehydration solution | **Maintained** |
| ENT-PULMONARY COUGH PROBLEMS |  |
| IP: Pholcodine | **Refused** |
| IP: Mucolytic drugs, mucokinetic drugs, or helicidine before two years of age | **Reworded** |
| IP: Alimemazine, oxomemazine, promethazine | **Refused** |
| IP: Terpene-based suppositories | **Refused** |
| OP: Failure to propose a whooping cough booster vaccine for adults who are likely to become parents in the coming months or years (only applicable if the previous vaccination was more than 10 years ago). This booster vaccination should also be proposed to the family and entourage of expectant parents (parents, grand-parents, nannies/child minders) | **Refused** |
| BRONCHIOLITIS IN INFANTS |  |
| IP: Beta2 agonists, corticosteroids to treat an infant’s first case of bronchiolitis | **Maintained** |
| IP: H1-antagonists, cough suppressants, mucolytic drugs, or ribavirin to treat bronchiolitis | **Maintained** |
| IP: Antibiotics in the absence of signs indicating a bacterial infection (acute otitis media, fever, etc.) | **Maintained** |
| OP: 0.9% NaCl to relieve nasal congestion (not applicable if nasal congestion is already being treated with 3% NaCl delivered by a nebulizer) | **Maintained** |
| OP: Palivizumab in the following cases: (1) babies born both at less than 35 weeks of gestation and less than six months prior to the onset of a seasonal RSV epidemic; (2) children less than two years old who have received treatment for bronchopulmonary dysplasia in the past six months; (3) children less than two years old suffering from congenital heart disease with hemodynamic abnormalities | **Refused** |
| ENT INFECTIONS |  |
| IP: An antibiotic other than amoxicillin as a first-line treatment for acute otitis media, strep throat, or sinusitis (provided that the patient is not allergic to amoxicillin). An effective dose of amoxicillin for an pneumoncoccal infection is 80-90 mg/kg/day and an effective dose for a streptococcal infection is 50 mg/kg/day | **Reworded** |
| IP: Antibiotic treatment for a sore throat, without a positive rapid diagnostic test result, in children less than three years old | **Maintained** |
| IP: Antibiotics for -nasopharyngitis-congestive otitis-sore throat before three years of age-laryngitis;-as a first-line treatment for acute otitis media showing few symptoms, before two years of age | **Maintained** |
| IP: Antibiotics to treat otitis media with effusion (OME), except in the case of hearing loss or if OME lasts for more than three months | **Maintained** |
| IP: Corticosteroids to treat acute suppurative otitis media, nasopharyngitis, or strep throat | **Maintained** |
| IP: Nasal or oral decongestant (oxymetazoline, pseudoephedrine, naphazoline, ephedrine, tuaminoheptane, phenylephrine) | **Reworded** |
| IP: H1-antagonists with sedative or atropine-like effects (pheniramine, chlorpheniramine), or camphor; inhalers, nasal sprays, or suppositories containing menthol (or any terpene derivatives) before 30 months of age | **Maintained** |
| IP: Ethanolamine tenoate and other nasal antiseptics | **Refused** |
| IP: Ear drops in the case of acute otitis media | **Reworded** |
| OP: Doses in mg for drinkable (solutions of) amoxicillin or josamycin | **Refused** |
| OP: Acetaminophen/paracetamol combined with antibiotic treatment for ear infections to relieve pain | **Maintained** |
| ASTHMA |  |
| IP: Ketotifen and other H1-antagonists, sodium cromoglycate | **Maintained** |
| IP: Cough suppressants | **Maintained** |
| OP: Asthma inhaler appropriate for the child’s age | **Maintained** |
| OP: Preventative treatment (inhaled corticosteroids) in the case of persistent asthma | **Maintained** |
| DERMATOLOGICAL ACNE VULGARIS PROBLEMS |  |
| IP: Minocycline | **Maintained** |
| IP: Isotretinoin in combination with a member of the tetracycline family of antibiotics | **Maintained** |
| IP: The combined use of an oral and a local antibiotic | **Reworded** |
| IP: Oral or local antibiotics as a monotherapy (not in combination with another drug) | **Reworded** |
| IP: Cyproterone + ethinylestradiol as a contraceptive to allow isotretinoin per os | **Maintained** |
| IP: Androgenic progestins (levonorgestrel, norgestrel, norethisterone, lynestrenol, dienogest, contraceptive implants or vaginal rings) | **Maintained** |
| OP: Contraception (provided with a logbook/diary) for menstruating girls taking isotretinoin | **Maintained** |
| OP: Topical treatment (benzoyl peroxide, retinoids, or both) in combination with antibiotic therapy | **Maintained** |
| SCABIES |  |
| IP: The application of benzyl benzoate for periods longer than eight hours for infants and 12 hours for children or for pregnant girls | **Refused** |
| OP: A second dose of ivermectin two weeks after the first | **Reworded** |
| OP: Decontamination of household linen and clothes and treatment for other family members | **Maintained** |
| LICE |  |
| IP: The use of aerosols for infants, children with asthma, or children showing asthma- like symptoms such as dyspnea | **Refused** |
| RINGWORM |  |
| IP: Treatment other than griseofulvin for Microsporum | **Refused** |
| OP: Topical treatment combined with an orally-administered treatment | **Maintained** |
| OP: Griseofulvin taken during a meal containing a moderate amount of fat | **Refused** |
| IMPETIGO |  |
| IP: The combination of locally applied and orally administered antibiotic | **Maintained** |
| IP: Fewer than two applications per day for topical antibiotics | **Refused** |
| IP: Any antibiotic other than mupirocin as a first-line treatment (except in cases of hypersensitivity to mupirocin) | **Refused** |
| HERPES SIMPLEX |  |
| IP: Topical agents containing corticosteroids | **Maintained** |
| IP: Topical agents containing acyclovir before six years of age | **Refused** |
| OP: Acetaminophen/paracetamol during an outbreak of herpes | **Maintained** |
| OP: Orally administered acyclovir to treat primary herpetic gingivostomatitis | **Reworded** |
| ATOPIC ECZEMA |  |
| IP: A strong dermocorticoid (clobetasol propionate 0.05% Dermoval, betamethasone dipropionate Diprosone) applied to the face, the armpits or groin, and the backside of babies or young children. | **Reworded** |
| IP: More than one application per day of a dermocorticoid, except in cases of severe lichenification | **Maintained** |
| IP: Local or systemic antihistamine during the treatment of outbreaks | **Maintained** |
| IP: Topically applied 0.03% tacrolimus before two years of age | **Maintained** |
| IP: Topically applied 0.1% tacrolimus before 16 years of age | **Maintained** |
| IP: Oral corticosteroids to treat outbreaks | **Maintained** |
| NEUROPSYCHIATRIC EPILEPSY DISORDERS |  |
| IP: Carbamazepine, gabapentin, oxcarbazepine, phenytoin, pregabalin, tiagabine, or vigabatrin in the case of myoclonic epilepsy | **Maintained** |
| IP: Carbamazepine, gabapentin, oxcarbazepine, phenytoin, pregabaline, tiagabine, or vigabatrin in the case of epilepsy with absence seizures (especially for childhood absence epilepsy or juvenile absence epilepsy) | **Maintained** |
| IP: Levetiracetam, oxcarbamazepine in mL or in mg without systematically writing XX mg per Y mL | **Maintained** |
| DEPRESSION |  |
| IP: An SSRI antidepressant other than fluoxetine as a first-line treatment (in the case of pharmacotherapy) | **Maintained** |
| IP: Tricyclic antidepressants to treat depression | **Maintained** |
| NOCTURNAL ENURESIS |  |
| IP: Desmopressin administered by a nasal spray. | **Maintained** |
| IP: Desmopressin in the case of daytime symptoms | **Maintained** |
| IP: An anticholinergic agent used as a monotherapy in the absence of daytime symptoms | **Maintained** |
| IP: Tricyclic agents in combination with anticholinergic agents | **Maintained** |
| IP: Tricyclic agents as a first-line treatment | **Maintained** |
| ANOREXIA |  |
| IP: Cyproheptadine, clonidine | **Maintained** |
| ATTENTION DEFICIT DISORDER WITH OR WITHOUT HYPERACTIVITY |  |
| IP: Pharmacological treatment before age six (before school), except in severe cases | **Maintained** |
| IP: Antipsychotic drugs to treat attention deficit disorder without hyperactivity | **Maintained** |
| IP: Slow release methylphenidate as two doses per day, rather than only one dose | **Maintained** |
| OP: Recording a growth chart (height and weight) if the patient is taking methylphenidate | **Maintained** |

**Table B: Sensitivity analysis after the second round.** Differences in the results of the second round with and without the sensitivity analysis. Six experts where lost-to-follow up after the first round. The sensitivity analysis represents the median score and the agreement if the participation rate was 100%. To do that, the responses of the six experts lost-to-follow up were replaced by their first round answers. Moreover, in the second round the agreement was restricted to 85% instead of 75%.

|  | Second round with n=14 experts | | After sensitivity analysis, n=20 experts | |
| --- | --- | --- | --- | --- |
| Items | Median | Agreement (%) | Median | Agreement (%) |
| DIVERSE PAIN AND FEVER ILLNESSES |  |  |  |  |
| IP: Rectal administration of paracetamol as a first-line treatment | 8 | 78,9 | 8 | 80 |
| OP: Failure to give an osmotic laxative to patients being treated with morphine for a period of more than 48 hours | 8 | 82,4 | 8,5 | 80 |
| MOSQUITOS |  |  |  |  |
| IP: Citronella (lemon grass) essential oil | 9 | 82,4 | 9 | 80 |
| DIGESTIVE NAUSEA, VOMITING OR GASTROESOPHAGEAL REFLUX PROBLEMS |  |  |  |  |
| IP: Domperidone | 8 | 77,8 | 8 | 75 |
| OP: Oral rehydration solution | 9 | 100 | 90 | 80 |
| ENT INFECTIONS |  |  |  |  |
| IP: Antibiotic treatment for a sore throat, without a positive rapid diagnostic test result, in children less than three years old | 9 | 88,9 | 9 | 80 |
| ASTHMA |  |  |  |  |
| IP: Ketotifen and other H1-antagonists, sodium cromoglycate | 9 | 78,9 | 9 | 80 |
| NOCTURNAL ENURESIS |  |  |  |  |
| IP: Tricyclic agents in combination with anticholinergic agents | 9 | 89,5 | 9 | 80 |
| ANOREXIA |  |  |  |  |
| IP: Cyproheptadine, clonidine | 9 | 88,9 | 9 | 80 |
| DEPRESSION |  |  |  |  |
| IP: An SSRI antidepressant other than fluoxetine as a first-line treatment (in the case of pharmacotherapy) | 9 | 84,2 | 9 | 90 |
| DERMATOLOGICAL ACNE VULGARIS PROBLEMS |  |  |  |  |
| OP: Topical treatment (benzoyl peroxide, retinoids, or both) in combination with antibiotic therapy | 9 | 88,9 | 9 | 90 |

**Table C: Individual responses of each expert (n=73 items) –** (NA=Non available)

| DIVERSE PAIN AND FEVER ILLNESSES | **1st round** | | **2nd round** | |
| --- | --- | --- | --- | --- |
| (1.1) IP: Prescription of two alternating antipyretics as a first-line treatment | **Expert 1** | **Expert 2** | **Expert 1** | **Expert 2** |
| China | 9 | NA | NA | NA |
| Brazil | 9 | 9 | NA | NA |
| Belgium | 9 | NA | 9 | NA |
| Turkey | 9 | 9 | 9 | 9 |
| Vietnam | 9 | 9 | 9 | 9 |
| Ivory Coast | 9 | NA | 9 | NA |
| Portugal | 9 | 9 | 9 | 7 |
| Switzerland | 9 | 9 | 9 | 9 |
| England | 8 | 9 | NA | 9 |
| Ireland | 5 | NA | NA | NA |
| Malaysia | 9 | 8 | 9 | 9 |
| Canada | 1 | 8 | NA | 8 |
| (1.2) IP: Prescription of a medication other than acetaminophen/paracetamol as a first line treatment (except in the case of migraine) |  |  |  |  |
| China | 9 | NA | NA | NA |
| Brazil | 9 | 7 | NA | NA |
| Belgium | 9 | NA | 9 | NA |
| Turkey | 9 | 9 | 9 | 9 |
| Vietnam | 9 | 9 | 9 | 9 |
| Ivory Coast | 7 | NA | 8 | NA |
| Portugal | 9 | 9 | 9 | 7 |
| Switzerland | 9 | 9 | 9 | 9 |
| England | 8 | 8 | NA | 9 |
| Ireland | 9 | NA | NA | NA |
| Malaysia | 9 | 8 | 9 | 8 |
| Canada | 6 | 2 | NA | 3 |
| (1.3) IP: Rectal administration of paracetamol as a first-line treatment |  |  |  |  |
| China | 8 | NA | NA | NA |
| Brazil | 1 | 9 | NA | NA |
| Belgium | 7 | NA | 7 | NA |
| Turkey | 9 | 9 | 9 | 9 |
| Vietnam | 8 | 6 | 8 | 6 |
| Ivory Coast | 9 | NA | 8 | NA |
| Portugal | 7 | 1 | 8 | 1 |
| Switzerland | 9 | 9 | 9 | 9 |
| England | 8 | 9 | NA | 9 |
| Ireland | 5 | NA | NA | NA |
| Malaysia | 9 | 8 | 9 | 8 |
| Canada | 7 | 3 | NA | 9 |
| (1.4) IP: The combined use of two NSAIDs |  |  |  |  |
| China | 9 | NA | NA | NA |
| Brazil | 9 | 9 | NA | NA |
| Belgium | 9 | NA | 9 | NA |
| Turkey | 9 | 9 | 9 | 9 |
| Vietnam | 9 | 9 | 9 | 9 |
| Ivory Coast | 9 | NA | 9 | NA |
| Portugal | 9 | 9 | 9 | 9 |
| Switzerland | 9 | 9 | 9 | 9 |
| England | 9 | 9 | NA | 9 |
| Ireland | 9 | NA | NA | NA |
| Malaysia | 5 | 9 | 7 | 9 |
| Canada | 1 | 9 | NA | 9 |
| (1.6) IP: Opiates to treat migraine attacks |  |  |  |  |
| China | 8 | NA | NA | NA |
| Brazil | 9 | 9 | NA | NA |
| Belgium | 9 | NA | 9 | NA |
| Turkey | 9 | 9 | 9 | 9 |
| Vietnam | 9 | 9 | 9 | 9 |
| Ivory Coast | 9 | NA | 9 | NA |
| Portugal | 8 | 9 | 8 | 9 |
| Switzerland | 9 | 9 | 9 | 9 |
| England | 6 | 9 | NA | 9 |
| Ireland | 9 | NA | 9 | NA |
| Malaysia | 9 | 9 | 9 | 9 |
| Canada | 3 | 7 | NA | 8 |
| (1.7) OP: Failure to give sugar solution to new-born babies and infants under four months old two minutes prior to venipuncture |  |  |  |  |
| China | 6 | NA | NA | NA |
| Brazil | 9 | 9 | NA | NA |
| Belgium | 9 | NA | 9 | NA |
| Turkey | 9 | 9 | 9 | 9 |
| Vietnam | 9 | 9 | 9 | 9 |
| Ivory Coast | 8 | NA | 8 | NA |
| Portugal | 9 | 9 | 9 | 9 |
| Switzerland | 5 | 9 | 9 | 9 |
| England | 8 | 9 | NA | 9 |
| Ireland | 9 | NA | NA | NA |
| Malaysia | 6 | 2 | 8 | 8 |
| Canada | 8 | 8 | NA | 9 |
| (1.8) OP: Failure to give an osmotic laxative to patients being treated with morphine for a period of more than 48 hours |  |  |  |  |
| China | 6 | NA | NA | NA |
| Brazil | 5 | 9 | NA | NA |
| Belgium | 9 | NA | 9 | NA |
| Turkey | 9 | 9 | 9 | 9 |
| Vietnam | 9 | 9 | 9 | 9 |
| Ivory Coast | 9 | NA | 9 | NA |
| Portugal | 8 | 9 | 8 | 9 |
| Switzerland | 5 | 9 | 8 | 9 |
| England | 8 | 9 | NA | 9 |
| Ireland | 5 | NA | NA | NA |
| Malaysia | 6 | 2 | 8 | 8 |
| Canada | 6 | 8 | NA | 8 |
| URINARY INFECTIONS |  |  |  |  |
| (2.3) IP: Antibiotic prophylaxis following an initial infection without complications (except in the case of uropathy) |  |  |  |  |
| China | 9 | NA | NA | NA |
| Brazil | 9 | 9 | NA | NA |
| Belgium | 9 | NA | 9 | NA |
| Turkey | 9 | 9 | 9 | 9 |
| Vietnam | 9 | 9 | 9 | 9 |
| Ivory Coast | 9 | NA | 9 | NA |
| Portugal | 9 | 9 | 9 | 9 |
| Switzerland | 9 | 9 | 9 | 9 |
| England | 9 | 8 | NA | 9 |
| Ireland | 9 | NA | NA | NA |
| Malaysia | 9 | 8 | 9 | 9 |
| Canada | 1 | 8 | NA | 9 |
| (2.4) IP: Antibiotic prophylaxis in the case of asymptomatic bacterial infection (except in the case of uropathy) |  |  |  |  |
| China | 9 | NA | NA | NA |
| Brazil | 9 | 9 | NA | NA |
| Belgium | 9 | NA | 9 | NA |
| Turkey | 9 | 9 | 9 | 9 |
| Vietnam | 9 | 8 | 9 | 8 |
| Ivory Coast | 9 | NA | 9 | NA |
| Portugal | 9 | 9 | 9 | 9 |
| Switzerland | 9 | 9 | 9 | 9 |
| England | 6 | 8 | NA | 9 |
| Ireland | 9 | NA | NA | NA |
| Malaysia | 9 | 8 | 9 | 9 |
| Canada | 1 | 9 | NA | 9 |
| VITAMIN SUPPLEMENTS AND ANTIBIOTIC PROPHYLAXIS |  |  |  |  |
| (3.1) IP: Fluoride supplements prior to six months of age |  |  |  |  |
| China | 9 | NA | NA | NA |
| Brazil | 5 | 9 | NA | NA |
| Belgium | 9 | NA | 9 | NA |
| Turkey | 9 | 9 | 9 | 9 |
| Vietnam | 9 | 9 | 9 | 9 |
| Ivory Coast | 9 | NA | 9 | NA |
| Portugal | 9 | 9 | 9 | 9 |
| Switzerland | 9 | 9 | 9 | 9 |
| England | 8 | 9 | NA | 9 |
| Ireland | 9 | NA | NA | NA |
| Malaysia | 9 | 9 | 9 | 9 |
| Canada | 1 | 8 | NA | 9 |
| MOSQUITOS |  |  |  |  |
| (4.2) IP: Citronella (lemon grass) essential oil |  |  |  |  |
| China | 1 | NA | NA | NA |
| Brazil | 9 | 1 | NA | NA |
| Belgium | 7 | NA | 8 | NA |
| Turkey | 1 | 1 | 1 | 1 |
| Vietnam | 9 | 9 | 9 | 9 |
| Ivory Coast | 7 | NA | 7 | NA |
| Portugal | 7 | 9 | 7 | 9 |
| Switzerland | 9 | 9 | 9 | 9 |
| England | NA | 9 | NA | 9 |
| Ireland | 9 | NA | NA | NA |
| Malaysia | 5 | 9 | 7 | 9 |
| Canada | 9 | 7 | NA | 8 |
| (4.3) IP:  Anti-insect bracelets to protect against mosquitos and ticks |  |  |  |  |
| China | 9 | NA | NA | NA |
| Brazil | 9 | 9 | NA | NA |
| Belgium | 9 | NA | 9 | NA |
| Turkey | 1 | 1 | 1 | 1 |
| Vietnam | 9 | 9 | 9 | 9 |
| Ivory Coast | 9 | NA | 9 | NA |
| Portugal | 9 | 5 | 9 | 9 |
| Switzerland | 9 | 9 | 9 | 9 |
| England | 8 | 9 | NA | 9 |
| Ireland | 9 | NA | 9 | NA |
| Malaysia | 5 | 8 | 7 | 9 |
| Canada | 2 | 9 | NA | 9 |
| (4.4) IP:  Ultrasonic pest control devices, vitamin B1, homeopathy, electric bug zappers, sticky tapes without insecticide |  |  |  |  |
| China | 9 | NA | NA | NA |
| Brazil | 9 | 9 | NA | NA |
| Belgium | 9 | NA | 9 | NA |
| Turkey | 1 | 1 | 1 | 1 |
| Vietnam | 9 | 9 | 9 | 9 |
| Ivory Coast | 9 | NA | 9 | NA |
| Portugal | 8 | 9 | 8 | 9 |
| Switzerland | 9 | 9 | 9 | 9 |
| England | NA | 9 | NA | 9 |
| Ireland | 8 | NA | NA | NA |
| Malaysia | 5 | 7 | 9 | 9 |
| Canada | 2 | 8 | NA | 9 |
| (4.7) OP: Mosquito nets and clothes treated with pyrethroids |  |  |  |  |
| China | 8 | NA | NA | NA |
| Brazil | 9 | 9 | NA | NA |
| Belgium | 9 | NA | 9 | NA |
| Turkey | 1 | 1 | 1 | 1 |
| Vietnam | 9 | 9 | 9 | 9 |
| Ivory Coast | 3 | NA | 7 | NA |
| Portugal | 8 | 9 | 8 | 9 |
| Switzerland | 9 | 9 | 9 | 9 |
| England | NA | 9 | NA | 9 |
| Ireland | 9 | NA | NA | NA |
| Malaysia | 9 | 3 | 9 | 8 |
| Canada | 9 | 8 | NA | 9 |
| DIGESTIVE NAUSEA, VOMITING OR GASTROESOPHAGEAL REFLUX PROBLEMS |  |  |  |  |
| (5.1) IP: Metoclopramide |  |  |  |  |
| China | 9 | NA | NA | NA |
| Brazil | 9 | 9 | NA | NA |
| Belgium | 3 | NA | 3 | NA |
| Turkey | 9 | 9 | 7 | 9 |
| Vietnam | 9 | 9 | 9 | 9 |
| Ivory Coast | 9 | NA | 9 | NA |
| Portugal | 9 | 9 | 9 | 9 |
| Switzerland | 9 | NA | 9 | 9 |
| England | 8 | 9 | NA | 9 |
| Ireland | 5 | NA | NA | NA |
| Malaysia | 8 | 8 | 8 | 9 |
| Canada | 5 | 7 | NA | 8 |
| (5.2) IP: Domperidone |  |  |  |  |
| China | 8 | NA | NA | NA |
| Brazil | 9 | 9 | NA | NA |
| Belgium | 3 | NA | 3 | NA |
| Turkey | 9 | 9 | 8 | 9 |
| Vietnam | 8 | 6 | 9 | 6 |
| Ivory Coast | 9 | NA | 9 | NA |
| Portugal | 9 | 9 | 9 | 9 |
| Switzerland | 7 | 9 | 8 | 9 |
| England | 5 | NA | NA | 9 |
| Ireland | 9 | NA | NA | NA |
| Malaysia | 2 | 8 | 8 | 8 |
| Canada | 5 | 8 | NA | 8 |
| (5.3) IP: Oral administration of an intravenous proton pump inhibitor (notably by nasogastric tube) |  |  |  |  |
| China | 9 | NA | NA | NA |
| Brazil | 9 | 9 | NA | NA |
| Belgium | 9 | NA | 9 | NA |
| Turkey | 9 | 9 | 9 | 9 |
| Vietnam | 9 | 9 | 9 | 9 |
| Ivory Coast | 8 | NA | 8 | NA |
| Portugal | 9 | 9 | 9 | 9 |
| Switzerland | 9 | 9 | 9 | 9 |
| England | 8 | 9 | NA | 9 |
| Ireland | 9 | NA | NA | NA |
| Malaysia | 8 | 9 | 8 | 9 |
| Canada | 1 | 8 | NA | 9 |
| (5.4) IP: Gastric antisecretory drugs to treat gastroesophageal reflux, dyspepsia, the crying of new-born babies (in the absence of any other signs or symptoms), as well as faintness in infants |  |  |  |  |
| China | 9 | NA | NA | NA |
| Brazil | 9 | NA | 9 | NA |
| Belgium | 9 | NA | 9 | NA |
| Turkey | 6 | 6 | 8 | 7 |
| Vietnam | 7 | 6 | 9 | 6 |
| Ivory Coast | 9 | NA | 9 | NA |
| Portugal | 8 | 9 | 8 | 9 |
| Switzerland | 9 | 9 | 9 | 9 |
| England | 7 | 9 | NA | 9 |
| Ireland | 9 | NA | NA | NA |
| Malaysia | 9 | 9 | 9 | 9 |
| Canada | 1 | 8 | NA | 9 |
| (5.5) IP: The combined use of proton pump inhibitors and NSAIDs, for a short period of time, in patients without risk factors |  |  |  |  |
| China | 9 | NA | NA | NA |
| Brazil | 9 | 9 | NA | NA |
| Belgium | 9 | NA | 9 | NA |
| Turkey | 9 | 9 | 9 | 9 |
| Vietnam | 9 | 9 | 9 | 9 |
| Ivory Coast | 9 | NA | 9 | NA |
| Portugal | 8 | 9 | 8 | 9 |
| Switzerland | 9 | 9 | 9 | 9 |
| England | 8 | 9 | NA | 9 |
| Ireland | 9 | NA | NA | NA |
| Malaysia | 9 | 8 | NA | 9 |
| Canada | 3 | 9 | NA | 9 |
| (5.6) IP: The use of type H2 antihistamines for long periods of treatment |  |  |  |  |
| China | 8 | NA | NA | NA |
| Brazil | 9 | 9 | NA | NA |
| Belgium | 9 | NA | 9 | NA |
| Turkey | 9 | 9 | 9 | 9 |
| Vietnam | 9 | 9 | 9 | 9 |
| Ivory Coast | 9 | NA | 9 | NA |
| Portugal | 8 | 9 | 8 | 9 |
| Switzerland | 9 | 9 | 9 | 9 |
| England | NA | 8 | NA | 9 |
| Ireland | 8 | NA | NA | NA |
| Malaysia | 7 | 9 | NA | 9 |
| Canada | 4 | 7 | NA | 8 |
| (5.9) OP: Oral rehydration solution |  |  |  |  |
| China | 9 | NA | NA | NA |
| Brazil | 9 | 9 | NA | NA |
| Belgium | 9 | NA | 9 | NA |
| Turkey | 9 | 9 | 5 | 6 |
| Vietnam | 9 | 9 | 9 | 9 |
| Ivory Coast | 9 | NA | 9 | NA |
| Portugal | 9 | 9 | 9 | 9 |
| Switzerland | 9 | 9 | 9 | 9 |
| England | 8 | 9 | NA | 9 |
| Ireland | 7 | NA | NA | NA |
| Malaysia | 9 | 9 | NA | 9 |
| Canada | 9 | 9 | NA | 9 |
| DIARRHEA |  |  |  |  |
| (6.1) IP: Loperamide before 3 years of age |  |  |  |  |
| China | 9 | NA | NA | NA |
| Brazil | 9 | NA | 9 | NA |
| Belgium | 7 | NA | 9 | NA |
| Turkey | 6 | 6 | 6 | 9 |
| Vietnam | 9 | 9 | 9 | 9 |
| Ivory Coast | 9 | NA | 9 | NA |
| Portugal | 9 | 9 | 9 | 9 |
| Switzerland | 8 | 9 | 8 | 9 |
| England | 8 | 8 | NA | 9 |
| Ireland | 9 | NA | NA | NA |
| Malaysia | 9 | 9 | 9 | 9 |
| Canada | 1 | 8 | NA | 9 |
| (6.2) IP: Loperamide in the case of invasive diarrhea |  |  |  |  |
| China | 8 | NA | NA | NA |
| Brazil | 9 | 9 | NA | NA |
| Belgium | 9 | NA | 9 | NA |
| Turkey | 9 | 9 | 9 | 9 |
| Vietnam | 9 | 9 | 9 | 9 |
| Ivory Coast | 9 | NA | 9 | NA |
| Portugal | 9 | 9 | 9 | 9 |
| Switzerland | 9 | 9 | 9 | 9 |
| England | 8 | 9 | NA | 9 |
| Ireland | 9 | NA | NA | NA |
| Malaysia | 9 | 9 | 9 | 9 |
| Canada | 1 | 9 | NA | 9 |
| (6.4) IP: The use of Saccharomyces boulardii in powder form, or in a capsule that has to be opened prior to ingestion, to treat patients with a central venous catheter or an immunodeficiency |  |  |  |  |
| China | 9 | NA | NA | NA |
| Brazil | 9 | 9 | NA | NA |
| Belgium | 9 | NA | 9 | NA |
| Turkey | 9 | 9 | 9 | 9 |
| Vietnam | 9 | 9 | 9 | 9 |
| Ivory Coast | 8 | NA | 8 | NA |
| Portugal | 9 | 9 | 9 | 9 |
| Switzerland | 9 | 9 | 9 | 9 |
| England | 2 | 9 | NA | 9 |
| Ireland | 9 | NA | NA | NA |
| Malaysia | 8 | 9 | 9 | 9 |
| Canada | 1 | 8 | NA | 9 |
| (6.5) IP: Intestinal antiseptics |  |  |  |  |
| China | 8 | NA | NA | NA |
| Brazil | 9 | 9 | NA | NA |
| Belgium | 9 | NA | 9 | NA |
| Turkey | 9 | 9 | 9 | 9 |
| Vietnam | 9 | 9 | 9 | 9 |
| Ivory Coast | 9 | NA | 9 | NA |
| Portugal | 9 | 9 | 9 | 9 |
| Switzerland | 9 | 9 | 9 | 9 |
| England | NA | 9 | NA | 9 |
| Ireland | 9 | NA | NA | NA |
| Malaysia | 9 | 9 | 9 | 9 |
| Canada | 1 | 8 | NA | 9 |
| (6.6) OP: Oral rehydration solution |  |  |  |  |
| China | 9 | NA | NA | NA |
| Brazil | 9 | 9 | NA | NA |
| Belgium | 9 | NA | 9 | NA |
| Turkey | 9 | 9 | 6 | 6 |
| Vietnam | 9 | 9 | 9 | 9 |
| Ivory Coast | 9 | NA | 9 | NA |
| Portugal | 9 | 9 | 9 | 9 |
| Switzerland | 9 | 9 | 9 | 9 |
| England | 8 | 9 | NA | 9 |
| Ireland | 1 | NA | NA | NA |
| Malaysia | NA | 8 | 9 | 9 |
| Canada | 9 | 9 | NA | 9 |
| ENT-PULMONARY COUGH PROBLEMS |  |  |  |  |
| (7.2) IP: Mucolytic drugs, mucokinetic drugs, or helicidine before two years of age |  |  |  |  |
| China | 9 | NA | NA | NA |
| Brazil | 5 | 7 | NA | NA |
| Belgium | 9 | NA | 9 | NA |
| Turkey | 9 | 9 | 9 | 9 |
| Vietnam | 9 | 9 | 9 | 9 |
| Ivory Coast | 9 | NA | 9 | NA |
| Portugal | 7 | 9 | 8 | 9 |
| Switzerland | 9 | 9 | 9 | 9 |
| England | 9 | 1 | NA | 1 |
| Ireland | 9 | NA | NA | NA |
| Malaysia | 8 | 2 | 8 | 8 |
| Canada | 9 | 9 | NA | 9 |
| BRONCHIOLITIS IN INFANTS |  |  |  |  |
| (8.1) IP: Beta2 agonists, corticosteroids to treat an infant’s first case of bronchiolitis |  |  |  |  |
| China | 9 | NA | NA | NA |
| Brazil | 9 | 9 | NA | NA |
| Belgium | 9 | NA | 9 | NA |
| Turkey | 9 | 9 | 9 | 9 |
| Vietnam | 6 | 9 | 6 | 9 |
| Ivory Coast | 9 | NA | 9 | NA |
| Portugal | 9 | 9 | 9 | 9 |
| Switzerland | 9 | 9 | 9 | 9 |
| England | 9 | 9 | NA | 9 |
| Ireland | 9 | NA | NA | NA |
| Malaysia | 9 | 9 | 9 | 9 |
| Canada | 1 | 8 | NA | 9 |
| (8.2) IP: H1-antagonists, cough suppressants, mucolytic drugs, or ribavirin to treat bronchiolitis |  |  |  |  |
| China | 9 | NA | NA | NA |
| Brazil | 9 | 9 | NA | NA |
| Belgium | 9 | NA | 9 | NA |
| Turkey | 9 | 9 | 9 | 9 |
| Vietnam | 9 | 9 | 9 | 9 |
| Ivory Coast | 9 | NA | 9 | NA |
| Portugal | 9 | 9 | 9 | 9 |
| Switzerland | 9 | 9 | 9 | 9 |
| England | 9 | 9 | NA | 9 |
| Ireland | 9 | NA | NA | NA |
| Malaysia | 9 | 9 | 9 | 9 |
| Canada | 9 | 9 | NA | 9 |
| (8.3) IP: Antibiotics in the absence of signs indicating a bacterial infection (acute otitis media, fever, etc.) |  |  |  |  |
| China | 9 | NA | NA | NA |
| Brazil | 9 | 9 | NA | NA |
| Belgium | 9 | NA | 9 | NA |
| Turkey | 9 | 9 | 9 | 9 |
| Vietnam | 9 | 9 | 9 | 9 |
| Ivory Coast | 9 | NA | 9 | NA |
| Portugal | 9 | 9 | 9 | 9 |
| Switzerland | 9 | 9 | 9 | 9 |
| England | 9 | 9 | NA | 9 |
| Ireland | 9 | NA | NA | NA |
| Malaysia | 9 | 9 | 9 | 9 |
| Canada | 9 | 9 | NA | 9 |
| (8.4) OP: 0.9% NaCl to relieve nasal congestion (not applicable if nasal congestion is already being treated with 3% NaCl delivered by a nebulizer) |  |  |  |  |
| China | 9 | NA | NA | NA |
| Brazil | 9 | 9 | NA | NA |
| Belgium | 9 | NA | 9 | NA |
| Turkey | 9 | 9 | 9 | 9 |
| Vietnam | 9 | 9 | 9 | 9 |
| Ivory Coast | 9 | NA | 9 | NA |
| Portugal | 9 | 9 | 9 | 9 |
| Switzerland | 9 | 9 | 9 | 9 |
| England | 2 | 9 | NA | 9 |
| Ireland | 9 | NA | NA | NA |
| Malaysia | 8 | 9 | 8 | 9 |
| Canada | 1 | 9 | NA | 9 |
| ENT INFECTIONS |  |  |  |  |
| (9.1) IP: An antibiotic other than amoxicillin as a first-line treatment for acute otitis media, strep throat, or sinusitis (provided that the patient is not allergic to amoxicillin). An effective dose of amoxicillin for an pneumoncoccal infection is 80-90 mg/kg/day and an effective dose for a streptococcal infection is 50 mg/kg/day |  |  |  |  |
| China | 8 | NA | NA | NA |
| Brazil | 9 | 9 | NA | NA |
| Belgium | 9 | NA | 9 | NA |
| Turkey | 9 | 9 | 9 | 9 |
| Vietnam | 9 | 9 | 9 | 9 |
| Ivory Coast | 9 | NA | 9 | NA |
| Portugal | 9 | 9 | 9 | 9 |
| Switzerland | 7 | 9 | 7 | 9 |
| England | 6 | 8 | NA | 9 |
| Ireland | 9 | NA | NA | NA |
| Malaysia | 5 | 8 | 7 | 9 |
| Canada | 9 | 9 | NA | 9 |
| (9.2) IP: Antibiotic treatment for a sore throat, without a positive rapid diagnostic test result, in children less than three years old |  |  |  |  |
| China | 9 | NA | NA | NA |
| Brazil | 9 | 9 | NA | NA |
| Belgium | 9 | NA | 9 | NA |
| Turkey | 9 | 9 | 9 | 9 |
| Vietnam | 9 | 9 | 9 | 9 |
| Ivory Coast | 9 | NA | 9 | NA |
| Portugal | 8 | 9 | 8 | 9 |
| Switzerland | 9 | 9 | 9 | 9 |
| England | 3 | 9 | NA | 9 |
| Ireland | 4 | NA | NA | NA |
| Malaysia | 5 | 8 | 5 | 9 |
| Canada | 1 | 8 | NA | 9 |
| (9.3) IP: Antibiotics for -nasopharyngitis-congestive otitis-sore throat before three years of age-laryngitis;-as a first-line treatment for acute otitis media showing few symptoms, before two years of age |  |  |  |  |
| China | 9 | NA | NA | NA |
| Brazil | 9 | 9 | NA | NA |
| Belgium | 9 | NA | 9 | NA |
| Turkey | 9 | 9 | 9 | 9 |
| Vietnam | 9 | 9 | 9 | 9 |
| Ivory Coast | 9 | NA | 9 | NA |
| Portugal | 7 | 9 | 8 | 9 |
| Switzerland | 9 | 9 | 9 | 9 |
| England | 7 | 9 | NA | 9 |
| Ireland | 3 | NA | NA | NA |
| Malaysia | 5 | 4 | 9 | 8 |
| Canada | 3 | 4 | NA | 5 |
| (9.4) IP: Antibiotics to treat otitis media with effusion (OME), except in the case of hearing loss or if OME lasts for more than three months |  |  |  |  |
| China | 9 | NA | NA | NA |
| Brazil | 9 | 9 | NA | NA |
| Belgium | 9 | NA | 9 | NA |
| Turkey | 9 | 9 | 9 | 9 |
| Vietnam | 9 | 9 | 9 | 9 |
| Ivory Coast | 9 | NA | 9 | NA |
| Portugal | 7 | 9 | 7 | 9 |
| Switzerland | 9 | 9 | 9 | 9 |
| England | 6 | 9 | NA | 9 |
| Ireland | 1 | NA | NA | NA |
| Malaysia | 8 | 2 | 9 | 8 |
| Canada | 1 | 8 | NA | 9 |
| (9.5) IP: Corticosteroids to treat acute suppurative otitis media, nasopharyngitis, or strep throat |  |  |  |  |
| China | 9 | NA | NA | NA |
| Brazil | 9 | 9 | NA | NA |
| Belgium | 9 | NA | 9 | NA |
| Turkey | 9 | 9 | 9 | 9 |
| Vietnam | 6 | 9 | 6 | 8 |
| Ivory Coast | 9 | NA | 9 | NA |
| Portugal | 9 | NA | 9 | 9 |
| Switzerland | 9 | 9 | 9 | 9 |
| England | 8 | 9 | NA | 9 |
| Ireland | 9 | NA | NA | NA |
| Malaysia | 8 | 9 | 6 | 9 |
| Canada | 1 | 9 | NA | 9 |
| (9.6) IP: Nasal or oral decongestant (oxymetazoline, pseudoephedrine, naphazoline, ephedrine, tuaminoheptane, phenylephrine) |  |  |  |  |
| China | 8 | NA | NA | NA |
| Brazil | 9 | 7 | NA | NA |
| Belgium | 9 | NA | 9 | NA |
| Turkey | 6 | 6 | 9 | 9 |
| Vietnam | 9 | 9 | 9 | 9 |
| Ivory Coast | 9 | NA | 9 | NA |
| Portugal | 8 | 5 | 9 | 9 |
| Switzerland | 9 | 9 | 9 | 9 |
| England | 8 | 9 | NA | 9 |
| Ireland | 9 | NA | NA | NA |
| Malaysia | 5 | 8 | 5 | 9 |
| Canada | 4 | 6 | NA | 8 |
| (9.7) IP: H1-antagonists with sedative or atropine-like effects (pheniramine, chlorpheniramine), or camphor; inhalers, nasal sprays, or suppositories containing menthol (or any terpene derivatives) before 30 months of age |  |  |  |  |
| China | 8 | NA | NA | NA |
| Brazil | 5 | 9 | NA | NA |
| Belgium | 9 | NA | 9 | NA |
| Turkey | 9 | 9 | 9 | 9 |
| Vietnam | 9 | 9 | 9 | 9 |
| Ivory Coast | 9 | NA | 9 | NA |
| Portugal | 7 | 9 | 8 | 9 |
| Switzerland | 9 | 9 | 9 | 9 |
| England | 9 | 9 | NA | 9 |
| Ireland | 9 | NA | NA | NA |
| Malaysia | 8 | 9 | 9 | 9 |
| Canada | 1 | 9 | NA | 9 |
| (9.9) IP: Ear drops in the case of acute otitis media |  |  |  |  |
| China | 8 | NA | NA | NA |
| Brazil | 9 | 9 | NA | NA |
| Belgium | 7 | NA | 7 | NA |
| Turkey | 9 | 9 | 9 | 9 |
| Vietnam | 9 | 9 | 9 | 9 |
| Ivory Coast | 8 | NA | 8 | NA |
| Portugal | 9 | 9 | 9 | 9 |
| Switzerland | 9 | 9 | 9 | 9 |
| England | 8 | 9 | NA | 9 |
| Ireland | 5 | NA | NA | NA |
| Malaysia | 9 | 9 | 9 | 9 |
| Canada | 7 | 7 | NA | 8 |
| (9.11) OP: Acetaminophen/paracetamol combined with antibiotic treatment for ear infections to relieve pain |  |  |  |  |
| China | 9 | NA | NA | NA |
| Brazil | 9 | 9 | NA | NA |
| Belgium | 9 | NA | 9 | NA |
| Turkey | 9 | 9 | 9 | 9 |
| Vietnam | 9 | 9 | 9 | 9 |
| Ivory Coast | 9 | NA | 9 | NA |
| Portugal | 9 | 9 | 9 | 9 |
| Switzerland | 5 | 9 | 9 | 9 |
| England | 7 | 9 | NA | 9 |
| Ireland | 9 | NA | NA | NA |
| Malaysia | 9 | 9 | 9 | 9 |
| Canada | 1 | 9 | NA | 9 |
| ASTHMA |  |  |  |  |
| (10.1) IP: Ketotifen and other H1-antagonists, sodium cromoglycate |  |  |  |  |
| China | 9 | NA | NA | NA |
| Brazil | 6 | 9 | NA | NA |
| Belgium | 9 | NA | 9 | NA |
| Turkey | 9 | 9 | 9 | 9 |
| Vietnam | 9 | 9 | 9 | 9 |
| Ivory Coast | 9 | NA | 9 | NA |
| Portugal | 7 | 9 | 7 | 9 |
| Switzerland | 9 | 9 | 9 | 9 |
| England | 8 | 9 | NA | 9 |
| Ireland | 5 | NA | NA | NA |
| Malaysia | 1 | 9 | 5 | 9 |
| Canada | 2 | 9 | NA | 9 |
| (10.2) IP: Cough suppressants |  |  |  |  |
| China | 8 | NA | NA | NA |
| Brazil | 9 | 9 | NA | NA |
| Belgium | 9 | NA | 9 | NA |
| Turkey | 9 | 9 | 9 | 9 |
| Vietnam | 9 | 9 | 9 | 9 |
| Ivory Coast | 9 | NA | 9 | NA |
| Portugal | 9 | 9 | 9 | 9 |
| Switzerland | 9 | 9 | 9 | 9 |
| England | 8 | 9 | NA | 9 |
| Ireland | 9 | NA | NA | NA |
| Malaysia | 9 | 9 | NA | 9 |
| Canada | 1 | 9 | NA | 9 |
| (10.3) OP: Asthma inhaler appropriate for the child’s age |  |  |  |  |
| China | 9 | NA | NA | NA |
| Brazil | 9 | 9 | NA | NA |
| Belgium | 9 | NA | 9 | NA |
| Turkey | 9 | 9 | 9 | NA |
| Vietnam | 9 | 9 | 9 | 9 |
| Ivory Coast | 9 | NA | 9 | NA |
| Portugal | 9 | 9 | 9 | 9 |
| Switzerland | 9 | 9 | 9 | 9 |
| England | 8 | 9 | NA | 9 |
| Ireland | 9 | NA | NA | NA |
| Malaysia | 9 | 9 | 9 | 9 |
| Canada | 9 | 9 | NA | 9 |
| (10.4) OP: Preventative treatment (inhaled corticosteroids) in the case of persistent asthma |  |  |  |  |
| China | 8 | NA | NA | NA |
| Brazil | 9 | 9 | NA | NA |
| Belgium | 9 | NA | 9 | NA |
| Turkey | 9 | 9 | 9 | NA |
| Vietnam | 9 | 9 | 9 | 9 |
| Ivory Coast | 9 | NA | 9 | NA |
| Portugal | 9 | 9 | 9 | 9 |
| Switzerland | 9 | NA | 9 | 9 |
| England | 9 | 9 | NA | 9 |
| Ireland | 7 | NA | NA | NA |
| Malaysia | 9 | 8 | 9 | 9 |
| Canada | 9 | 9 | NA | 9 |
| DERMATOLOGICAL ACNE VULGARIS PROBLEMS |  |  |  |  |
| (11.2) IP: Isotretinoin in combination with a member of the tetracycline family of antibiotics |  |  |  |  |
| China | 9 | NA | NA | NA |
| Brazil | 9 | 9 | NA | NA |
| Belgium | 9 | NA | 9 | NA |
| Turkey | 9 | 9 | 9 | 9 |
| Vietnam | 9 | 9 | 9 | 9 |
| Ivory Coast | 9 | NA | 9 | NA |
| Portugal | 7 | 9 | 7 | 9 |
| Switzerland | 9 | 9 | 9 | 9 |
| England | 8 | 9 | NA | 9 |
| Ireland | 5 | NA | NA | NA |
| Malaysia | NA | 9 | NA | 9 |
| Canada | NA | 9 | NA | 9 |
| (11.3) IP: The combined use of an oral and a local antibiotic |  |  |  |  |
| China | 9 | NA | NA | NA |
| Brazil | 9 | 9 | NA | NA |
| Belgium | 9 | NA | 9 | NA |
| Turkey | 9 | 9 | 9 | 9 |
| Vietnam | 9 | 9 | 9 | 9 |
| Ivory Coast | 9 | NA | 9 | NA |
| Portugal | 7 | 9 | 8 | 9 |
| Switzerland | 9 | 9 | 9 | 9 |
| England | 8 | 9 | NA | 9 |
| Ireland | 5 | NA | NA | NA |
| Malaysia | NA | 9 | NA | 9 |
| Canada | 9 | 8 | NA | 9 |
| (11.4) IP: Oral or local antibiotics as a monotherapy (not in combination with another drug) |  |  |  |  |
| China | 8 | NA | NA | NA |
| Brazil | 5 | 9 | NA | NA |
| Belgium | 9 | NA | 9 | NA |
| Turkey | 9 | 9 | 9 | 9 |
| Vietnam | 9 | 9 | 9 | 9 |
| Ivory Coast | 9 | NA | 9 | NA |
| Portugal | 8 | 9 | 8 | 9 |
| Switzerland | 9 | NA | 1 | 9 |
| England | 5 | 9 | NA | 9 |
| Ireland | 5 | NA | NA | NA |
| Malaysia | NA | 2 | NA | 8 |
| Canada | 9 | 8 | 9 | 9 |
| SCABIES |  |  |  |  |
| (12.2) OP: A second dose of ivermectin two weeks after the first |  |  |  |  |
| China | 8 | NA | NA | NA |
| Brazil | 5 | 9 | NA | NA |
| Belgium | 9 | NA | 9 | NA |
| Turkey | 1 | 1 | 1 | 1 |
| Vietnam | 9 | 9 | 9 | 9 |
| Ivory Coast | 7 | NA | 8 | NA |
| Portugal | 7 | 9 | 8 | 9 |
| Switzerland | 7 | 9 | 9 | 9 |
| England | 8 | 9 | NA | 9 |
| Ireland | 9 | NA | NA | NA |
| Malaysia | 8 | 9 | 9 | 9 |
| Canada | 9 | 7 | NA | 8 |
| (12.3) OP: Decontamination of household linen and clothes and treatment for other family members |  |  |  |  |
| China | 9 | NA | NA | NA |
| Brazil | 9 | 9 | NA | NA |
| Belgium | 9 | NA | 9 | NA |
| Turkey | 9 | 9 | 9 | 9 |
| Vietnam | 9 | 9 | 9 | 9 |
| Ivory Coast | 9 | NA | 9 | NA |
| Portugal | 9 | 9 | 9 | 9 |
| Switzerland | 9 | 9 | 9 | 9 |
| England | 8 | 8 | NA | 8 |
| Ireland | 9 | NA | NA | NA |
| Malaysia | 9 | 9 | 9 | 9 |
| Canada | 9 | 9 | NA | 9 |
| RINGWORM |  |  |  |  |
| (14.2) OP: Topical treatment combined with an orally-administered treatment |  |  |  |  |
| China | 9 | NA | NA | NA |
| Brazil | 9 | 9 | NA | NA |
| Belgium | 9 | NA | 9 | NA |
| Turkey | 9 | 9 | 9 | 9 |
| Vietnam | 9 | 9 | 9 | 9 |
| Ivory Coast | 8 | NA | 8 | NA |
| Portugal | 8 | 9 | 8 | 9 |
| Switzerland | 9 | 9 | 9 | 9 |
| England | 8 | 3 | NA | 3 |
| Ireland | 9 | NA | NA | NA |
| Malaysia | 8 | 8 | NA | 8 |
| Canada | 4 | 3 | NA | 9 |
| IMPETIGO |  |  |  |  |
| (15.1) IP: The combination of locally applied and orally administered antibiotic |  |  |  |  |
| China | 8 | NA | NA | NA |
| Brazil | 9 | 9 | NA | NA |
| Belgium | 7 | NA | 9 | NA |
| Turkey | 9 | 9 | 9 | 9 |
| Vietnam | 9 | 9 | 9 | 9 |
| Ivory Coast | 8 | NA | 8 | NA |
| Portugal | 9 | 9 | 9 | 9 |
| Switzerland | 9 | 9 | 9 | 9 |
| England | 6 | 9 | NA | 9 |
| Ireland | 8 | NA | NA | NA |
| Malaysia | 8 | 8 | NA | 8 |
| Canada | 9 | 6 | NA | 9 |
| HERPES SIMPLEX |  |  |  |  |
| (16.1) IP: Topical agents containing corticosteroids |  |  |  |  |
| China | 9 | NA | NA | NA |
| Brazil | 9 | 9 | NA | NA |
| Belgium | 9 | NA | 9 | NA |
| Turkey | 9 | 9 | 9 | 9 |
| Vietnam | 9 | 9 | 9 | 9 |
| Ivory Coast | 9 | NA | 9 | NA |
| Portugal | 9 | 9 | 9 | 9 |
| Switzerland | 9 | 9 | 9 | 9 |
| England | 8 | 9 | NA | 9 |
| Ireland | 9 | NA | NA | NA |
| Malaysia | 8 | 7 | NA | 9 |
| Canada | 1 | 9 | NA | 9 |
| (16.3) OP: Acetaminophen/paracetamol during an outbreak of herpes |  |  |  |  |
| China | 9 | NA | NA | NA |
| Brazil | 9 | 9 | NA | NA |
| Belgium | 9 | NA | 9 | NA |
| Turkey | 9 | 9 | 9 | 9 |
| Vietnam | 9 | 9 | 9 | 9 |
| Ivory Coast | 9 | NA | 9 | NA |
| Portugal | 8 | 9 | 8 | 9 |
| Switzerland | 9 | 9 | 9 | 9 |
| England | 6 | 5 | NA | 5 |
| Ireland | 5 | NA | NA | NA |
| Malaysia | 8 | 9 | NA | 9 |
| Canada | 9 | 8 | NA | 9 |
| (16.4) OP: Orally administered acyclovir to treat primary herpetic gingivostomatitis |  |  |  |  |
| China | 9 | NA | NA | NA |
| Brazil | 5 | 7 | NA | NA |
| Belgium | 9 | NA | 9 | NA |
| Turkey | 9 | 9 | 9 | 9 |
| Vietnam | 9 | 9 | 9 | 9 |
| Ivory Coast | 9 | NA | 9 | NA |
| Portugal | 9 | 9 | 9 | 9 |
| Switzerland | 9 | 1 | 9 | 9 |
| England | 6 | 9 | NA | 9 |
| Ireland | 8 | NA | NA | NA |
| Malaysia | 8 | 8 | NA | 8 |
| Canada | 9 | 3 | NA | 9 |
| ATOPIC ECZEMA |  |  |  |  |
| (17.1) IP: A strong dermocorticoid (clobetasol propionate 0.05% Dermoval, betamethasone dipropionate Diprosone) applied to the face, the armpits or groin, and the backside of babies or young children. |  |  |  |  |
| China | 8 | NA | NA | NA |
| Brazil | 9 | 9 | NA | NA |
| Belgium | 9 | NA | 9 | NA |
| Turkey | 9 | 9 | 9 | 9 |
| Vietnam | 9 | 9 | 9 | 9 |
| Ivory Coast | 9 | NA | 9 | NA |
| Portugal | 8 | 9 | 9 | 9 |
| Switzerland | 9 | 9 | 9 | 9 |
| England | 9 | 9 | NA | 9 |
| Ireland | 5 | NA | NA | NA |
| Malaysia | 8 | 9 | 9 | 9 |
| Canada | 4 | 9 | NA | 9 |
| (17.2) IP: More than one application per day of a dermocorticoid, except in cases of severe lichenification |  |  |  |  |
| China | 8 | NA | NA | NA |
| Brazil | 9 | 9 | NA | NA |
| Belgium | 9 | NA | 9 | NA |
| Turkey | 9 | 9 | 9 | 9 |
| Vietnam | 9 | 9 | 9 | 9 |
| Ivory Coast | 9 | NA | 9 | NA |
| Portugal | 8 | 5 | 8 | 9 |
| Switzerland | 5 | 9 | 9 | 9 |
| England | 7 | 9 | NA | 9 |
| Ireland | 1 | NA | NA | NA |
| Malaysia | 8 | 8 | 9 | 9 |
| Canada | 9 | 2 | NA | 2 |
| (17.3) IP: Local or systemic antihistamine during the treatment of outbreaks |  |  |  |  |
| China | 8 | NA | NA | NA |
| Brazil | 9 | 7 | NA | NA |
| Belgium | 9 | NA | 9 | NA |
| Turkey | 9 | 9 | 9 | 9 |
| Vietnam | 9 | 9 | 9 | 9 |
| Ivory Coast | 9 | NA | 9 | NA |
| Portugal | 6 | 5 | 6 | 9 |
| Switzerland | 9 | 9 | 9 | 9 |
| England | 8 | 9 | NA | 9 |
| Ireland | 1 | NA | NA | NA |
| Malaysia | 7 | 5 | 7 | 7 |
| Canada | 7 | 7 | NA | 8 |
| (17.4) IP: Topically applied 0.03% tacrolimus before two years of age |  |  |  |  |
| China | 7 | NA | NA | NA |
| Brazil | 9 | 9 | NA | NA |
| Belgium | 9 | NA | 9 | NA |
| Turkey | 9 | 9 | 9 | 9 |
| Vietnam | 9 | 9 | 9 | 9 |
| Ivory Coast | 9 | NA | 9 | NA |
| Portugal | 8 | 9 | 8 | 9 |
| Switzerland | 9 | 9 | 9 | 9 |
| England | 9 | 9 | NA | 9 |
| Ireland | 5 | NA | NA | NA |
| Malaysia | 7 | 4 | 8 | 7 |
| Canada | 9 | 6 | 7 | NA |
| (17.5) IP: Topically applied 0.1% tacrolimus before 16 years of age |  |  |  |  |
| China | 7 | NA | NA | NA |
| Brazil | 9 | 9 | NA | NA |
| Belgium | 9 | NA | 9 | NA |
| Turkey | 9 | 9 | 9 | 9 |
| Vietnam | 9 | 9 | 9 | 9 |
| Ivory Coast | 9 | NA | 9 | NA |
| Portugal | 8 | NA | 8 | 9 |
| Switzerland | 8 | 1 | 9 | 8 |
| England | 5 | 9 | NA | 9 |
| Ireland | 1 | NA | NA | NA |
| Malaysia | 7 | 4 | 8 | 7 |
| Canada | 9 | 4 | NA | 5 |
| (17.6) IP: Oral corticosteroids to treat outbreaks |  |  |  |  |
| China | 7 | NA | NA | NA |
| Brazil | 9 | 7 | NA | NA |
| Belgium | 9 | NA | 9 | NA |
| Turkey | 9 | 9 | 9 | 9 |
| Vietnam | 9 | 9 | 9 | 9 |
| Ivory Coast | 9 | NA | 9 | NA |
| Portugal | 4 | 5 | 5 | 9 |
| Switzerland | 7 | 9 | 8 | 9 |
| England | 9 | 9 | NA | 9 |
| Ireland | 5 | NA | NA | NA |
| Malaysia | 7 | 8 | 7 | 8 |
| Canada | 6 | 8 | NA | 7 |
| NEUROPSYCHIATRIC EPILEPSY DISORDERS |  |  |  |  |
| (18.1) IP: Carbamazepine, gabapentin, oxcarbazepine, phenytoin, pregabalin, tiagabine, or vigabatrin in the case of myoclonic epilepsy |  |  |  |  |
| China | 9 | NA | NA | NA |
| Brazil | 9 | 9 | NA | NA |
| Belgium | 9 | NA | 9 | NA |
| Turkey | 9 | 9 | 9 | 9 |
| Vietnam | 9 | 9 | 9 | 9 |
| Ivory Coast | 9 | NA | 9 | NA |
| Portugal | 9 | 9 | 9 | 9 |
| Switzerland | 9 | 9 | 9 | 9 |
| England | 6 | 9 | NA | 9 |
| Ireland | 5 | NA | NA | NA |
| Malaysia | 7 | 8 | NA | 9 |
| Canada | 1 | 9 | NA | 9 |
| (18.2) IP: Carbamazepine, gabapentin, oxcarbazepine, phenytoin, pregabaline, tiagabine, or vigabatrin in the case of epilepsy with absence seizures (especially for childhood absence epilepsy or juvenile absence epilepsy) |  |  |  |  |
| China | 9 | NA | NA | NA |
| Brazil | 9 | 9 | NA | NA |
| Belgium | 9 | NA | 9 | NA |
| Turkey | 9 | 9 | 9 | 9 |
| Vietnam | 9 | 9 | 9 | 9 |
| Ivory Coast | 9 | NA | 9 | NA |
| Portugal | 9 | 9 | 9 | 9 |
| Switzerland | 9 | 9 | 9 | 9 |
| England | 8 | 9 | NA | 9 |
| Ireland | 5 | NA | NA | NA |
| Malaysia | 7 | 8 | NA | 9 |
| Canada | 2 | 9 | NA | 9 |
| DEPRESSION |  |  |  |  |
| (19.2) IP: Tricyclic antidepressants to treat depression |  |  |  |  |
| China | 8 | NA | NA | NA |
| Brazil | 9 | 9 | NA | NA |
| Belgium | 7 | NA | 7 | NA |
| Turkey | 9 | 9 | 9 | 9 |
| Vietnam | 9 | 9 | 9 | 9 |
| Ivory Coast | 9 | NA | 9 | NA |
| Portugal | 8 | 9 | 8 | 9 |
| Switzerland | 9 | 9 | 9 | 9 |
| England | 8 | 9 | NA | 9 |
| Ireland | 9 | NA | NA | NA |
| Malaysia | 8 | 9 | 9 | 9 |
| Canada | 2 | 9 | NA | 9 |
| NOCTURNAL ENURESIS |  |  |  |  |
| (20.1) IP: Desmopressin administered by a nasal spray. |  |  |  |  |
| China | 8 | NA | NA | NA |
| Brazil | 9 | 7 | NA | NA |
| Belgium | 9 | NA | 9 | NA |
| Turkey | 9 | 9 | 9 | 9 |
| Vietnam | 9 | 9 | 9 | 9 |
| Ivory Coast | 9 | NA | 9 | NA |
| Portugal | 9 | 5 | 9 | 9 |
| Switzerland | 9 | 1 | 9 | 9 |
| England | 4 | 9 | NA | 9 |
| Ireland | 7 | NA | NA | NA |
| Malaysia | 5 | 7 | 7 | 7 |
| Canada | 8 | 8 | NA | 8 |
| (20.2) IP: Desmopressin in the case of daytime symptoms |  |  |  |  |
| China | 8 | NA | NA | NA |
| Brazil | 9 | 9 | NA | NA |
| Belgium | 9 | NA | 9 | NA |
| Turkey | 9 | 9 | 9 | 9 |
| Vietnam | 9 | 9 | 9 | 9 |
| Ivory Coast | 9 | NA | 9 | NA |
| Portugal | 8 | 9 | 8 | 9 |
| Switzerland | 9 | 9 | 9 | 9 |
| England | 8 | 9 | NA | 9 |
| Ireland | 5 | NA | NA | NA |
| Malaysia | 9 | 8 | 9 | 9 |
| Canada | 1 | 9 | NA | 9 |
| (20.3) IP: An anticholinergic agent used as a monotherapy in the absence of daytime symptoms |  |  |  |  |
| China | 8 | NA | NA | NA |
| Brazil | 9 | 9 | NA | NA |
| Belgium | 9 | NA | 9 | NA |
| Turkey | 9 | 9 | 9 | 9 |
| Vietnam | 9 | 9 | 9 | 9 |
| Ivory Coast | 9 | NA | 9 | NA |
| Portugal | 9 | 9 | 9 | 9 |
| Switzerland | 9 | 9 | 9 | 9 |
| England | 7 | 9 | NA | 9 |
| Ireland | 5 | NA | NA | NA |
| Malaysia | 9 | 9 | 9 | 9 |
| Canada | 1 | 7 | NA | 7 |
| (20.4) IP: Tricyclic agents in combination with anticholinergic agents |  |  |  |  |
| China | 9 | NA | NA | NA |
| Brazil | 9 | 9 | NA | NA |
| Belgium | 9 | NA | 9 | NA |
| Turkey | 9 | 9 | 9 | 9 |
| Vietnam | 9 | 6 | 9 | 6 |
| Ivory Coast | 9 | NA | 9 | NA |
| Portugal | 7 | 9 | 7 | 9 |
| Switzerland | 9 | 9 | 9 | 9 |
| England | 5 | 9 | NA | 9 |
| Ireland | 5 | NA | NA | NA |
| Malaysia | 7 | 8 | 9 | 9 |
| Canada | 1 | 8 | NA | 8 |
| (20.5) IP: Tricyclic agents as a first-line treatment |  |  |  |  |
| China | 9 | NA | NA | NA |
| Brazil | 9 | 9 | NA | NA |
| Belgium | 9 | NA | 9 | NA |
| Turkey | 9 | 9 | 9 | 9 |
| Vietnam | 9 | 9 | 9 | 9 |
| Ivory Coast | 9 | NA | 9 | NA |
| Portugal | 9 | 9 | 9 | 9 |
| Switzerland | 9 | NA | 9 | 9 |
| England | 8 | 9 | NA | 9 |
| Ireland | 9 | NA | NA | NA |
| Malaysia | 9 | 8 | 9 | 8 |
| Canada | 1 | 9 | NA | 9 |
| ANOREXIA |  |  |  |  |
| (21.1) IP: Cyproheptadine, clonidine |  |  |  |  |
| China | 8 | NA | NA | NA |
| Brazil | 9 | 9 | NA | NA |
| Belgium | 5 | NA | 7 | NA |
| Turkey | 9 | 9 | 9 | 9 |
| Vietnam | 9 | 9 | 9 | 9 |
| Ivory Coast | 9 | NA | 9 | NA |
| Portugal | 5 | 9 | 5 | 9 |
| Switzerland | 9 | 9 | 9 | 9 |
| England | 8 | 9 | NA | 9 |
| Ireland | 5 | NA | NA | NA |
| Malaysia | NA | 9 | NA | 9 |
| Canada | 2 | 8 | NA | 9 |
| ATTENTION DEFICIT DISORDER WITH OR WITHOUT HYPERACTIVITY |  |  |  |  |
| (22.1) IP: Pharmacological treatment before age six (before school), except in severe cases |  |  |  |  |
| China | 7 | NA | NA | NA |
| Brazil | 9 | 9 | NA | NA |
| Belgium | 9 | NA | 9 | NA |
| Turkey | 9 | 9 | 9 | 9 |
| Vietnam | 9 | 9 | 9 | 9 |
| Ivory Coast | 9 | NA | 9 | NA |
| Portugal | 9 | 9 | 9 | 9 |
| Switzerland | 9 | 9 | 9 | 9 |
| England | 9 | 9 | NA | 9 |
| Ireland | 9 | NA | NA | NA |
| Malaysia | 9 | 9 | 9 | 9 |
| Canada | 2 | 8 | NA | 8 |
| (22.2) IP: Antipsychotic drugs to treat attention deficit disorder without hyperactivity |  |  |  |  |
| China | 9 | NA | NA | NA |
| Brazil | 9 | 9 | NA | NA |
| Belgium | 9 | NA | 9 | NA |
| Turkey | 9 | 9 | 9 | 9 |
| Vietnam | 9 | 9 | 9 | 9 |
| Ivory Coast | 9 | NA | 9 | NA |
| Portugal | 8 | 9 | 9 | 9 |
| Switzerland | 5 | 9 | 9 | 9 |
| England | 8 | 9 | NA | 9 |
| Ireland | 9 | NA | NA | NA |
| Malaysia | 9 | 8 | 9 | 9 |
| Canada | 1 | 8 | NA | 8 |
| (22.3) IP: Slow release methylphenidate as two doses per day, rather than only one dose |  |  |  |  |
| China | 9 | NA | NA | NA |
| Brazil | 9 | 9 | NA | NA |
| Belgium | 9 | NA | 9 | NA |
| Turkey | 9 | 9 | 9 | 9 |
| Vietnam | 9 | 9 | 9 | 9 |
| Ivory Coast | 9 | NA | 9 | NA |
| Portugal | 9 | 9 | 9 | 9 |
| Switzerland | 9 | 9 | 9 | 9 |
| England | 8 | 9 | NA | 9 |
| Ireland | 7 | NA | NA | NA |
| Malaysia | 7 | 9 | 7 | 9 |
| Canada | 1 | 9 | NA | 9 |
| (22.4) OP: Recording a growth chart (height and weight) if the patient is taking methylphenidate |  |  |  |  |
| China | 8 | NA | NA | NA |
| Brazil | 9 | 9 | NA | NA |
| Belgium | 9 | NA | 9 | NA |
| Turkey | 9 | 9 | 9 | 9 |
| Vietnam | 9 | 9 | 9 | 9 |
| Ivory Coast | 9 | NA | 9 | NA |
| Portugal | 9 | 9 | 9 | 9 |
| Switzerland | 9 | 9 | 9 | 9 |
| England | 8 | 9 | NA | 9 |
| Ireland | 8 | NA | NA | NA |
| Malaysia | 8 | 9 | 9 | 9 |
| Canada | 1 | 9 | NA | 9 |
